# Fast response of fungal and prokaryotic communities to climate change manipulation in two contrasting tundra soils

**DOI:** 10.1186/s40793-019-0344-4

**Published:** 2019-09-18

**Authors:** Jana Voříšková, Bo Elberling, Anders Priemé

**Affiliations:** 1 Center for Permafrost (CENPERM), Department of Geosciences and Natural Resource Management, University of Copenhagen, Copenhagen, Denmark; 2 Department of Geochemistry, Geological Survey of Denmark and Greenland (GEUS), Copenhagen, Denmark; 3 Ecology Department, Climate and Ecosystem Sciences, Lawrence Berkeley National Laboratory, Berkeley, CA USA; 4 Department of Biology, University of Copenhagen, Copenhagen, Denmark

**Keywords:** Microbial communities, Climate warming, Enhanced precipitation, Snow fence, Microbial ecology, Fungi, Bacteria, Arctic soil, Greenland

## Abstract

**Background:**

Climate models predict substantial changes in temperature and precipitation patterns across Arctic regions, including increased winter precipitation as snow in the near future. Soil microorganisms are considered key players in organic matter decomposition and regulation of biogeochemical cycles. However, current knowledge regarding their response to future climate changes is limited. Here, we explore the short-term effect of increased snow cover on soil fungal, bacterial and archaeal communities in two tundra sites with contrasting water regimes in Greenland. In order to assess seasonal variation of microbial communities, we collected soil samples four times during the plant-growing season.

**Results:**

The analysis revealed that soil microbial communities from two tundra sites differed from each other due to contrasting soil chemical properties. Fungal communities showed higher richness at the dry site whereas richness of prokaryotes was higher at the wet tundra site. We demonstrated that fungal and bacterial communities at both sites were significantly affected by short-term increased snow cover manipulation. Our results showed that fungal community composition was more affected by deeper snow cover compared to prokaryotes. The fungal communities showed changes in both taxonomic and ecological groups in response to climate manipulation. However, the changes were not pronounced at all sampling times which points to the need of multiple sampling in ecosystems where environmental factors show seasonal variation. Further, we showed that effects of increased snow cover were manifested after snow had melted.

**Conclusions:**

We demonstrated rapid response of soil fungal and bacterial communities to short-term climate manipulation simulating increased winter precipitation at two tundra sites. In particular, we provide evidence that fungal community composition was more affected by increased snow cover compared to prokaryotes indicating fast adaptability to changing environmental conditions. Since fungi are considered the main decomposers of complex organic matter in terrestrial ecosystems, the stronger response of fungal communities may have implications for organic matter turnover in tundra soils under future climate.

**Electronic supplementary material:**

The online version of this article (10.1186/s40793-019-0344-4) contains supplementary material, which is available to authorized users.

## Introduction

Arctic tundra represents a globally significant biome covering 7.3 million km^2^ (~ 5% of Earth’s land area). It is characterized by low temperatures, a short plant-growing season and a long dark winter with sub-zero temperatures. In general, tundra soils contain high quantities of organic matter [47] due to the constrained decomposition processes caused by low temperatures and strong nutrient limitations [45]. This pool of organic matter can play an important role in the global terrestrial carbon (C) cycle if mineralized [76]. Microorganisms are the main drivers of degradation of soil organic C (SOC mineralization) into greenhouse gases [48, 83]. Global climate changes, including warming and altered precipitation, are predicted to be the most pronounced at northern latitudes [12, 50]. Therefore, the response of tundra soil microorganisms to these changes will have important consequences for ecosystem functioning and climate change feedbacks.

Nutrient cycling and the decomposition of organic matter in various habitats are driven by a diverse group of microorganisms (fungi, bacteria, archaea, and microeukaryotes). Fungi are considered the main decomposers in terrestrial ecosystems in part due to their production of a wide range of lignocellulytic enzymes with abilities to attack complex parts of soil organic matter [84]. Bacteria and archaea, unlike fungi, are rather involved in earlier stages of organic matter decomposition and a faster consumption of simple carbon compounds [60]. Evidence suggests that soil temperature and moisture are important environmental parameters affecting microbial activity [11, 79] and SOC decomposition rates [39, 75]. Climate change in cold regions may thus change the activity of soil microbial communities and boost their ability to decompose SOC, which may alter C storage in Arctic soils and influence CO_2_ concentration in the atmosphere [14].

Climate change is projected to increase winter precipitation as snow in northern latitudes during the coming decades [50]. The depth and duration of seasonal snow cover seem to be important parameters affecting microorganisms in tundra soil [17, 74] and thus SOC decomposition. The snow pack serves as an insulation layer for soil and vegetation [33], protecting the soil from extremely low air temperatures that occur during the Arctic winter. Deeper snow cover enhances the isolation effect, which rises winter soil temperature [59] leading to increased microbial activity [74]. The influence of snow cover on soil ecosystems is not limited to the winter season and can persist until late summer by affecting e.g. soil moisture, the length of plant growing season, or nutrient availability [24, 87, 90, 95]. Increased snow cover has been shown to affect tundra soil respiration [59, 94], density of shrub cover [62, 95], litter decomposition [13], and nutrient dynamics [74, 77] suggesting that deeper snow pack may potentially alter microbial communities. These findings are supported by a recent study by Xue et al. [96] reporting increased abundance of microbial functional genes involved in SOC decomposition as a result of deeper snow cover in tundra soil. Several recent studies assessing the effect of increased snow cover on microbial communities involved only specific groups of microbes [20, 57, 61]. However, to better understand and quantify tundra soil ecosystem processes, it is essential to address both fungal and prokaryotic communities at the same time. Also, most studies are performed following several years of in situ ecosystem manipulation [58, 73, 78] and, thus, do not investigate fast (within one-two years) and immediate responses of tundra soil microbial communities to enhanced snow cover. In contrast to long-term climate manipulation treatments, where the ecological drivers of microbial communities are mostly changes in soil chemical properties and/or vegetation cover, the changes of microbial communities following short-term treatment are mainly associated with habitat loss and opening of new niches that may be colonized by rapidly responding microbes [9, 30, 38, 67]. Since the mechanisms driving microbial communities are different for short- and long-term climate manipulation experiments, both types of manipulation treatments need to be studied. Today, soil microbial communities form one of the largest uncertainties to climate model predictions [36]. Thus, understanding the short-term effect of increased winter precipitation on the dynamics and functioning of soil microbial communities, major decomposers of SOC, is necessary for predicting if Arctic tundra soils become a sink or source of CO_2_ under future climates.

The main aim of this study was to characterize compositional changes in fungal and prokaryotic communities in response to increased snow cover in two Arctic tundra soils with contrasting water regimes and vegetation types (a dry mixed-shrub heath and a wet fen, respectively). In order to mimic increased winter precipitation, we implemented a snow manipulation experiment using snow fences to trap drifting snow during winter on Disko Island, Western Greenland [13]. To our knowledge, this is the first study addressing short-term effects of increased winter precipitation simultaneously on fungal, bacterial and archaeal communities in tundra soil. The results of Wallenstein et al. [92] indicate that activity of extracellular enzymes in tundra soils varies with season and very limited information is available on temporal dynamics of microbial communities in these type of soils therefore we repeated soil sampling at four time points between June and October which is snow-free period in our study site. We hypothesized that microbial community diversity will be quickly and markedly affected by increased snow cover mirroring changes in soil moisture and nutrient availability induced by deepened snow and that this effect will also be manifest in the snow-free period. Furthermore, we hypothesized that the bacterial community composition and richness will be more affected by short-term snow-manipulation compared to the fungal community as bacteria generally have higher growth and turnover rates than fungi [80] and thus a potentially faster adaptation to the new conditions. Based on results from the same experimental site showing that deeper snow during the winter significantly increases the rate of litter decomposition [13] we hypothesized that snow manipulated plots will exhibit higher abundance of saprotrophic and plant pathogenic fungi compared to control sites.

## Methods

### Study site and experimental set-up

The study area was in the Blæsedalen valley (69 °16′N, 53°27′W) on Disko Island in Western Greenland. The area has low-arctic climate with a mean annual air temperature of − 3.0 °C, the warmest month is July with a mean temperature of 7.9 °C and the coldest month is March with a mean temperature − 14 °C (period 1991–2011). The mean annual amount of precipitation is ~ 400 mm of which ~ 40% fall in form of snow (1994–2006), measured at Arctic Station, approximately 3 km from the study area [46]. The study area lies within the discontinuous permafrost zone.

The snow manipulation experiment was established at two tundra sites approximately 200 m apart. The first site was dry mixed-shrub heath dominated by *Vaccinium uliginosum, Betula nana, Salix glauca*, *Empetrum nigrum,* and *Cassiope tetragona*, and soil consists of basaltic rock fragments covered by a thin (5–10 cm) organic horizon – indicated throughout the manuscript as the dry site (D). The second site was wet fen dominated by *Carex aquatilis ssp. stans*, *Carex rariflora, Eriophorum angustifolium*, *Paludella squarrosa, Tomentypnum nitens, Salix arctophila* and *Dryas octopetala*, and soil consists of basaltic rock fragments covered by a ~ 20-cm peat layer - indicated as the wet site (W). At both sites, the snow depth manipulation experiment included six replicate blocks providing sufficient statistical power. Each block contained a 14.7-m-long and 1.5-m-tall snow fence to create snowdrifts on the leeward (south) side of the fences during winter (indicated as snow manipulation - S), while the windward side of the snow fence represented ambient snow conditions (indicated as control - C). Snow depth on manipulated side of snow fences was on average 150 cm, snow depth on control side was on average 70 cm, and these conditions lasted for at least 3 months during the winter period. Snow fences were established in June 2012 and July 2013 at the dry site and the wet site, respectively. Soil temperatures (5 cm depth) were measured continuously in snow manipulation and control plots of three blocks, using TinyTag PB-5001 thermistor probes (Gemini Data Loggers, Chichester, UK) and logged every hour. The sites have been studied previously with respect to the effect of increased snow cover on litter decomposition [13], litter-decomposing fungi [20] and methane fluxes [27, 63].

### Sample collection and processing

Sampling of the topsoil (0–5 cm) at both sites was repeated four times during the entire snow-free period of 2014: June 23–26 (immediately after snow melt on both sides of snow fences), July 18–22, September 07–10, and October 10–12. Five soil cores (2 cm in diameter) were collected at control and manipulated side of each snow fence (six replicates at each site) at a distance of ca. 1 m from each other in a parallel line with the snow fence in a distance of ca. 2.5 m from the fence. Soil samples were processed within 24 h after sampling in the laboratory of nearby Arctic Station. The material from the five replicate soil cores was combined and homogenized and woody roots were removed. Subsamples for DNA isolation, chemical analyses and quantification of fungal biomass were frozen at − 20 °C, and DNA was isolated within 1 week in the laboratory of Arctic Station using Nucleospin Soil Kit (Macherey-Nagel, Düren, Germany). Three DNA extractions were performed from each sample and mixed afterwards. Approximately 0.25 g of soil was used for each DNA extraction. All samples were kept frozen in insulated boxes during the transportation to Copenhagen where they were stored at − 20 °C until further analysis.

### Soil properties and fungal biomass

Records of soil temperature at 5 cm soil depth were carried out using a portable thermometer (Spectrum Technologies, Aurora, IL, USA) [20]. Soil samples were weighed into tin capsules and analyzed for total C and N on Isoprime isotope ratio mass spectrometer (Elementar, Langenselbold, Germany) coupled to a Eurovector CN elemental analyzer (Eurovector, Pavia, Italy). Soil organic matter (SOM) was estimated through loss of ignition at 550 °C. Soil pH was measured by adding double deionised H_2_O to dry soil in a 1:10 ratio, and water content was calculated by freeze-drying of soil subsamples. Total ergosterol was extracted from 0.25 g of freeze-dried soil with 10% KOH in methanol and analyzed by high-performance liquid chromatography using a method modified from Bååth [7].

### Illumina amplicon sequencing of fungal and bacterial communities

For fungal community analysis, the primers gITS7/ITS4 [49] were used to amplify the ITS2 region of rRNA operon, and for prokaryotic community analysis, primer pair 515F/806R targeting the V4 region of 16S rRNA gene was used [19]. PCR amplifications were performed with primers containing template-specific sequences extended by a 2-nt linker and 4–6-nt barcode. Each of three independent 10 μL reactions per DNA sample contained 2 μL of 5x polymerase buffer, 1 μL of 10 mg mL^− 1^ bovine serum albumin, 0.5 μL of each primer (0.01 mM), 0.2 μL of dNTPs (10 mM), 0.1 μL of PCRBIO HiFi Polymerase (PCR Biosystems, London, UK), 0.5 μL of template DNA (DNA concentration 10–100 ng/μL), and 5.2 μL of H_2_0. The cycling conditions were 95 °C for 1 min; 35 cycles of 95 °C for 15 s; 56 °C for 20 s; and 72 °C for 20 s, followed by 72 °C for 5 min for primers gITS7/ITS4, and 95 °C for 1 min; 30 cycles of 95 °C for 15 s; 50 °C for 20 s; and 72 °C for 20 s, followed by 72 °C for 5 min for primers 515F/806R. PCR products from three PCR replicates were pooled and purified using HighPrep™ PCR clean up system (MAGBIO, Gaithersburg, MD, USA). The concentration of PCR products was quantified using the Qubit® 2.0 Fluorometer (Life Technologies, Carlsbad, CA, USA). PCR products were mixed equimolarly and Illumina adapter sequences were ligated on amplicons using TruSeq DNA PCR-Free LT Sample Prep Kit (Illumina, San Diego, CA, USA). The amplicon library was subjected to sequencing on Illumina MiSeq 2 × 250 bp paired-end platform in the National High-throughput DNA Sequencing Centre (Copenhagen, Denmark). Sequencing data are available in the MGRAST public database (http://metagenomics.anl.gov/, dataset numbers 4782666.3 and 4782667.3).

### Bioinformatic analyses

Illumina sequencing data were processed using the combination of pipelines proposed by Bálint et al. [8] and Větrovský and Baldrian [85]. Paired-end reads were merged using fastq-join [3]. The ITS region of fungal sequences was extracted using ITSx [10]. Sequences were clustered at 97% similarity level using UPARSE [32]. During the clustering chimeric sequences were discarded. Singleton sequences were removed from the dataset and consensus sequences were constructed for each operational taxonomic unit (OTU). Closest hits of fungal and bacterial sequences were identified using UNITE database [1] and SILVA reference database release 119 [72], respectively.

### Diversity and statistical analysis

The most abundant OTUs representing 80% of all fungal or prokaryotic sequences were used for calculation of diversity estimates, providing combined information on OTU richness and evenness in an individual samples [86]. These estimates were calculated based on datasets containing 9000 fungal and 2200 bacterial randomly selected sequences from each sample. OTU accumulation curves were calculated using R package vegan [64]. Venn diagrams, where OTUs with > 0.01% abundance were considered as present, were calculated using Venn Diagram Plotter (https://omics.pnl.gov/software/venn-diagram-plotter). The data were analyzed with a combination of constrained and unconstrained multivariate statistical methods in order to account for total variation in the data and variation explainable by the environmental data. Partial principal component analysis (PCA) where snow fence block was set as a covariate and redundancy analysis (RDA) with interactive forward selection and 999 Monte Carlo permutations were used to explain the variation in the data. Both analyses were performed in the multivariate data analysis software CANOCO 5.0 [82]. Significant community differences were tested by analysis of similarity (ANOSIM) with Bray-Curtis dissimilarity of relative abundances calculated using software PRIMER 6 [56]. Soil properties and microbial diversity were tested for differences using paired t-tests following square root transformation of the data. Statistically significant differences in abundance of OTUs or higher-level microbial taxa between control and manipulated sites were tested by DESeq2 with Benjamini-Hochberg correction [55, 93].

## Results

### Soil properties

The soil properties significantly differed between the dry and the wet sites. Water content averaged 53 and 80% (*P =* 6 × 10^− 18^), C content 20.1 and 26.8% (*P =* 6 × 10^− 7^), N content 0.60 and 1.46% (*P* = 2 × 10^− 16^), pH 5.15 and 5.9 (*P* = 1 × 10^− 21^) and organic matter content 39.3 and 57.3% (*P* = 1 × 10^− 10^) at the dry and wet site respectively (Additional file 1). However, we found no significant differences in soil chemical properties between control and snow manipulated plots at any of the two sites. Soil temperature (measured at 5 cm depth) was higher in snow-manipulated plots compare to control plots during the snow covered period and the difference was more pronounced at the dry site compared to the wet site (Additional file 2) [20]. The mean annual soil temperatures in 5 cm depth were 0.02 °C and 1.06 °C in the snow-manipulated plots and − 1.33 °C and 0.68 °C in the control plots at the dry and wet site respectively.

### Fungal communities

**Fig. 1:**
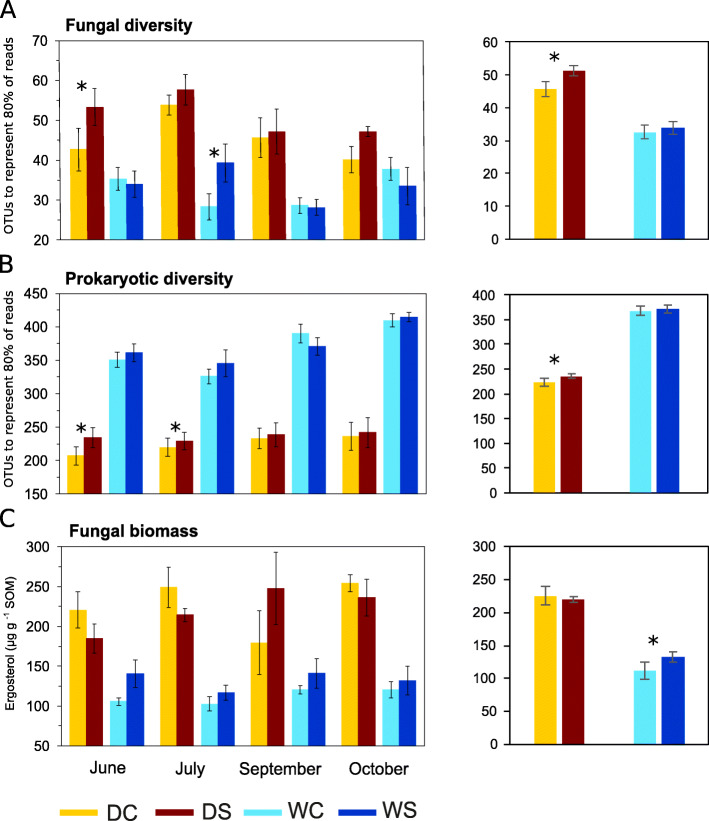
Fungal (**a**) and bacterial (**b**) diversity estimates and fungal biomass (ergosterol content) (**c**) in the dry (D) and the wet (W) tundra soil in control (C) and snow-manipulated (S) plots by season and as a seasonal average (column chart). Diversity is expressed as the number of the most abundant OTUs (operational taxonomic units), which represented 80% of all sequences. The data represent the means with standard errors (*n* = 6), for seasonal average (*n* = 24). Statistically significant effects (*P* < 0.05) of treatment between C and S at a specific site are indicated by asterisk

The analysis of fungal community was performed with 2,307,725 sequences that remained after quality filtering, and removal of chimeric and non-fungal sequences. An average of 24,039 sequences was obtained (minimum of 9324) per sample. OTU accumulation curves are shown in Additional file 3. Fungal diversity (expressed as the number of OTUs that represented 80% of all sequences in each sample calculated at 9000 sequences per sample) was significantly higher (*P* = 1 × 10^− 9^) at the dry site (48.5 ± 1.6) compared to the wet site (33.2 ± 1.3) (Fig. 1). Fungal diversity at the dry site was significantly lower (*P =* 0.032) in control plots (45.6 ± 2.2 OTUs representing 80% of sequences) compared to snow-manipulated plots (51.3 ± 2.1 OTUs representing 80% of sequences) with the most pronounced difference in June (*P =* 0.027). Similarly to fungal diversity, fungal biomass (expressed as ergosterol content) was substantially higher (*P* = 1 × 10^− 12^) at the dry site (223 ± 9.7 μg ergosterol g^− 1^ SOM) compared to the wet site (122 ± 4.6 μg ergosterol g^− 1^ SOM). Contrary to fungal diversity, fungal biomass showed significant differences (*P =* 0.031) at the wet site, where the biomass was the largest in snow-manipulated plots (Fig. 1).

**Fig. 2:**
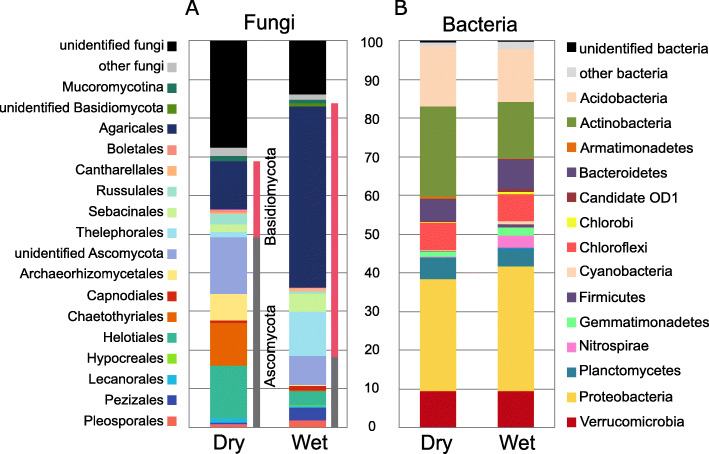
Phylogenetic assignment of fungal (**a**) and bacterial (**b**) sequences from the dry and the wet tundra (control sites only). The data represent the mean abundances from four time points (*n* = 24)

**Fig. 3:**
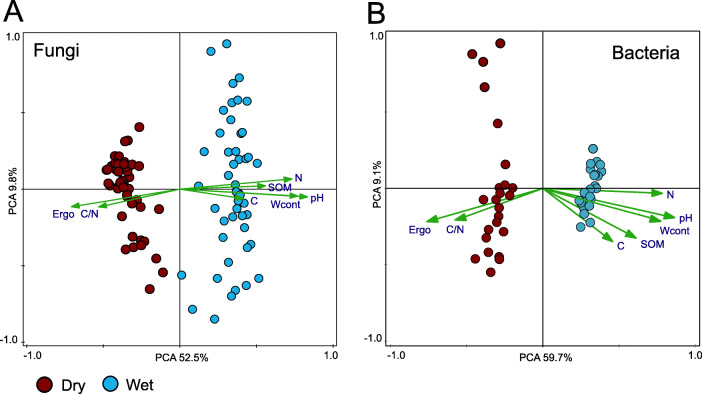
Principal component analysis (PCA) of relative abundances of fungal (**a**) and bacterial (**b**) genera from control plots and environmental variables. All genera with > 0.01% abundance in the dry or the wet tundra soil were considered. Ergo, ergosterol; N, nitrogen; C, carbon; SOM, soil organic matter; Wcont, water content

**Fig. 4:**
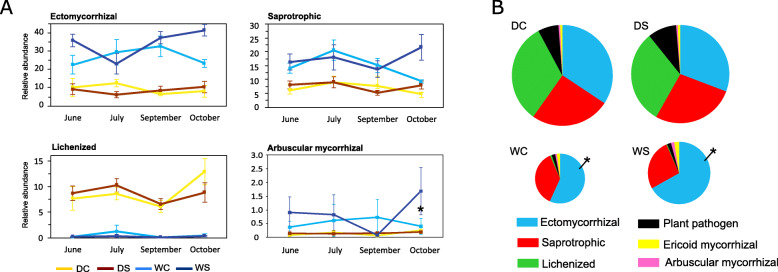
Proportion of sequences belonging to different fungal ecological groups in the dry (D) and the wet (W) tundra soil in control (C) and snow-manipulated (S) plots by season (**a**) and as a seasonal average (**b**) with the chart area corresponding to the ergosterol content (pie chart). The data represent the means with standard errors (*n* = 6), for seasonal average (*n* = 24). Statistically significant effects of treatment between C and S are indicated by asterisk (DESeq, Benjamini-Hochberg correction, *P* < 0.05)

**Fig. 5:**
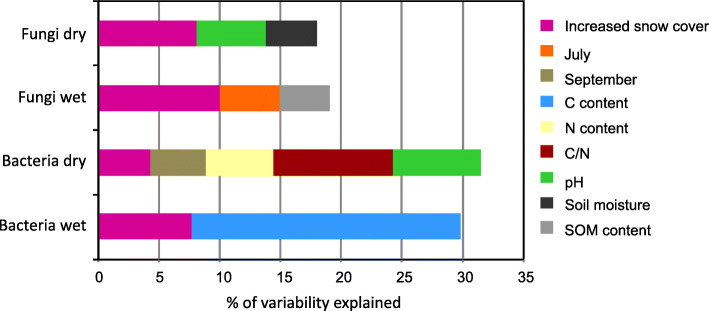
Results of redundancy analysis (RDA) of the 40 most abundant bacterial and fungal OTUs from the dry and the wet tundra soil. Depicted parameters had significant (*P* < 0.05) effect on variation of bacterial or fungal community. C (carbon), N (nitrogen), SOM (soil organic matter)

The overall fungal community was largely comprised of the *Basidiomycota* (41.6%) and *Ascomycota* (32.9%) (Fig. 2). *Mucoromycotina* comprised of 1.07% of the fungal community and detected *Glomeromycota* and *Chytridiomycota* represented less than 0.5% of the sequences. PCA (principal component analysis) of control plots from combined dataset of the dry and the wet sites showed that the two sites harbored very distinct fungal populations and that the difference was driven by the soil chemical properties (Fig. 3). Only 10% of fungal OTUs were present at both the dry site and the wet site (Additional file 4). Therefore, we analyzed the data from the two sites separately to be able to target the effect of increased snow cover on the fungal communities. At the the dry site, *Ascomycota* represented 49.6% of identified sequences whereas *Basidiomycota* represented only 20.4%. Here, the most abundant fungal orders were ascomycetous *Helotiales* (13.6%), *Chaetothyriales* (11.0%), *Archaeorhizomycetales* (6.9%) and basidiomycetous *Agaricales* (12.3%). Contrary to the dry site, the wet site contained distinctively more sequences belonging to *Basidiomycota* (66.2%) with order *Agaricales* representing almost half of all sequences; and *Ascomycota* showed lower relative abundance representing only 18.7% of identified fungal community (Fig. 2). Ectomycorrhizal, saprotrophic and lichenized fungi showed similar abundance at the dry site, whereas the wet site was dominated by the sequences belonging to ectomycorrhizal fungi (Fig. 4b). The effect of season on entire fungal community composition was rather minor (Fig. 5) therefore we analyzed the response of fungi to increased snow cover both for individual time points (*n* = 6) and for pooled samples across all time points (*n* = 24).

**Fig. 6:**
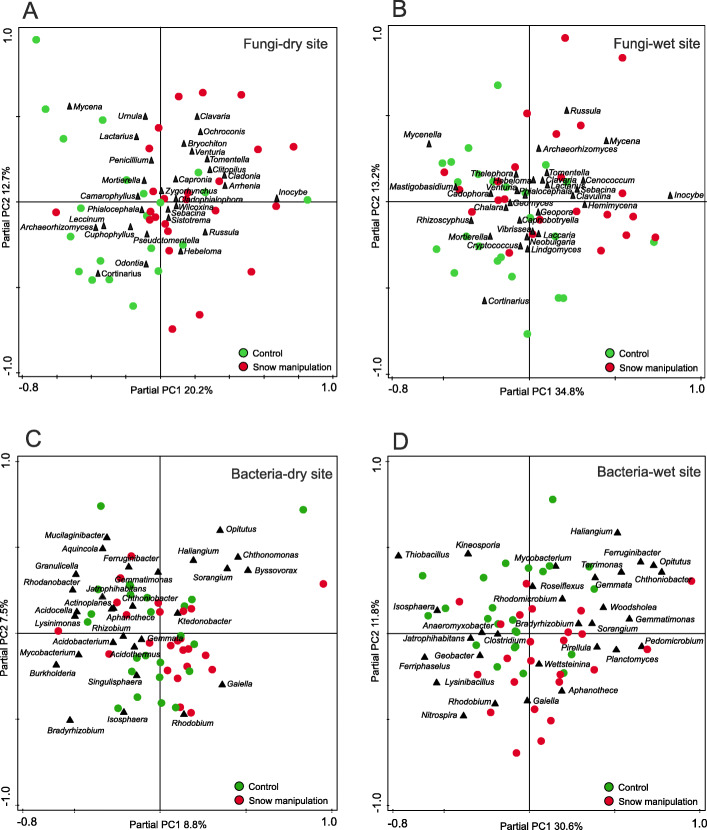
Partial principal component analysis (PCA) of relative abundances of the 30 most abundant fungal genera from the dry site (**a**) and the wet site (**b**) and bacterial genera from the dry site (**c**) and the wet site (**d**), snow fence block was set as a covariate

**Fig. 7:**
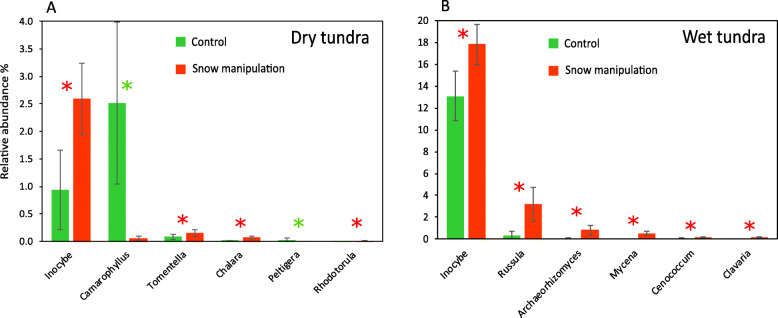
Relative abundance of the six most abundant fungal genera from the dry (**a**) and the wet (**b**) tundra soil with statistically significant difference between control (green) and snow-manipulated (red) plots across all seasons (DESeq2, Benjamini-Hochberg correction, *P* < 0.05). Green asterisk indicates significantly higher abundance in control plots, red asterisk indicates significantly higher abundance in snow-manipulated plots. The data represent the means with standard errors (*n* = 24)

The effect of increased-snow cover on fungal communities was profound: only 46% of fungal OTUs on the dry site and 38% on the wet site were shared between snow-manipulated plots and controls (Additional file 4), shared OTUs were represented by 96% of sequences. Six (dry site) and six (wet site) out of the thirty most abundant fungal OTUs showed statistically significant differences (*P* < 0.05) in the abundance between manipulated and control plots (Additional file 5) across all time points. The eighth most abundant OTU at the dry site assigned to lichenized *Lecanoromycetes* was significantly (*P =* 0.004) less abundant in manipulated plots (1.2%) compared to control plots (1.7%), whereas the second most abundant OTU from the wet site, the ectomycorrhizal *Inocybe*, had five times higher abundance (*P* = 4 × 10^− 12^) in increased-snow plots compared to control (Additional file 5 - data for seasonal average). At both sites, PCA showed a separation of fungal communities between the treated and control plots (Fig. 6) and the analysis of similarities (ANOSIM) based on Bray-Curtis distance revealed that the OTU composition was significantly different between the fungal communities found in ambient and manipulated plots at both sites (dry site R = 0.80, *P* = 0.01; wet site R = 0.72, *P* = 0.01). Results of redundancy analysis (RDA) of the 40 most abundant fungal OTUs showed that snow manipulation treatment had significant effect on the fungal communities and explained 8.1 and 10% of variability at the dry and the wet sites, respectively (Fig. 5). In total, 294 fungal genera were identified in the whole dataset. The most abundant fungal genera significantly affected by snow manipulation were *Inocybe* (*P =* 0.002), *Camarophyllus* (*P* = 3 × 10^− 7^)*, Tomentella* (*P =* 0.002)*, Chalara* (*P =* 0.002)*, Peltigera* (*P* = 6 × 10^− 12^) *and Rhodotorula* (*P =* 0.025) at the dry site and *Inocybe* (*P =* 0.049)*, Russula* (*P* = 7 × 10^− 6^)*, Archaeorhizomyces* (*P =* 0.019)*, Mycena* (*P* = 9 × 10^− 6^)*, Cenococcum* (*P =* 0.047) *and Clavaria* (*P =* 0.001) at the wet site (Fig. 7). Fungal communities showed temporal variation during the snow-free period in response to the snow-manipulation treatment (Additional file 6). Fungal community collected during October was the most affected by increased snow cover compared to other seasons (Fig. 4a, Additional files 5 and 6). Ectomycorrhizal fungi inhabiting the wet site showed significantly higher abundance (*P =* 0.001) in treated plots compared to control plots (Fig. 4b). Also, the wet site in October showed significant differences (*P =* 0.033) in abundance of arbuscular mycorrhizal fungi (Fig. 4a).

### Prokaryotic communities

The analysis of prokaryotic community was performed with 1,214,686 sequences that remained after quality filtering, and removal of chimeric sequences and sequences not belonging to prokaryotes. An average of 12,653 sequences was obtained (minimum of 3098) per sample. OTU accumulation curves are shown in Additional file 3. Bacterial diversity (expressed as the number of OTUs that represented 80% of sequences in each sample calculated at 2200 sequences per sample) was substantially lower (*P* = 5 × 10^− 23^) at the dry site (230 ± 5.7) compared to the wet site (370 ± 6.0). At the dry site, bacterial diversity showed significant changes (*P =* 0.022) between control (224 ± 7.9) and snow-manipulated (236 ± 8.1) plot with the most pronounced differences in June (*P =* 0.012) and July (*P =* 0.042) (Fig. 1).

The overall prokaryotic community was dominated by *Proteobacteria* (29.2%), *Actinobacteria* (17.7%) and *Acidobacteria* (14.2%). Alike the fungal communities, PCA showed highly dissimilar bacterial communities at dry and wet tundra sites (Fig. 3). Therefore, further analysis was done separately for the two sites. Approximately 30% of bacterial OTUs were present at both the dry site and the wet site (Additional file 4). *Protebacteria* constituted the most dominant phylum at both sites representing 28.9% at the dry site and 32.2% at the wet site. The relative abundance of *Actinobacteria* was 23.4% at the dry site and smaller at 14.6% at the wet site. OTUs assigned to bacteria involved in nitrification process, ammonia-oxidizing (*P* = 2 × 10^− 156^) and nitrite-oxidizing bacteria (*P* = 2 × 10^− 118^), showed distinctively higher abundance at the wet site compared to the dry site (Additional file 7). For example, nitrite-oxidizing *Nitrospira* showed ten times relative abundance difference between the dry and the wet site (Fig. 2). In contrast, OTUs assigned to nitrogen-fixing bacterial taxa (e.g. *Cyanobacteria*, *Rhizobium* and *Bradyrhizobium)* showed significantly higher abundance (*P* = 5 × 10^− 45^) at the dry site compared to the the wet site (Additional file 7). We did not observed any significant effect of season on entire prokaryotic community composition (Fig. 5) therefore we analyzed the response of prokaryotes to increased snow cover both for individual time points (*n* = 6) and for pooled samples across all time points (*n* = 24).

Bacterial communities were apparently less influenced by the increased snow cover than fungi. Snow-manipulated plots and control plots shared 57 and 60% of bacterial OTUs at the dry and the wet sites, respectively (Additional file 4), shared OTUs were represented by 98.5% of sequences. Significant differences in abundance between treated and control plots were detected for three out of the thirty most abundant bacterial OTUs, both for the dry and the wet sites (Additional file 8). At the dry site two OTUs assigned to the *Acidothermaceae* family showed significant decrease (*P =* 0.026 and *P =* 0.026) in their abundance in response to the treatment. Whereas at the wet site two OTUs assigned to the class *Acidobacteria* showed significantly positive response (*P =* 0.048 and *P =* 0.031) to increased snow cover. ANOSIM analysis revealed that the OTU composition was significantly different between the prokaryotic communities only at the dry site (R = 0.37, *P* = 0.01), and not at the wet site (R = 0.33, *P* = 0.07). RDA of the prokaryotic community indicated that snow manipulation treatment explained 4.6 and 7.4% of community variability at the dry and the wet site, respectively (Fig. 5). PCA of the bacterial communities at both sites showed less clear distinction between treatment and control compared to fungal communities (Fig. 6).

*Archaea* represented on average 0.6 and 2.3% of the prokaryotic community at the dry and the wet site, respectively. *Archaea* showed significantly higher relative abundance at the wet site compared to the dry site (*P* = 4 × 10^− 15^), however, we did not find any significant differences in their abundance between control and snow-manipulated plots (Additional file 9).

## Discussion

### Microbial communities of dry and wet tundra soil

Dry and wet tundra sites located 200 m apart showed pronounced differences in fungal and bacterial community structure and diversity consistent with differences in key soil chemical properties (Fig. 3), which is in accordance with previous observations from permafrost-affected soils [43, 99]. Besides abiotic soil properties, plant species/litter amount and quality affect tundra soil microbial communities [21, 91]. As different plant communities dominated the dry and the wet sites, we are unable to differentiate between the main drivers of the observed differences. Bacterial diversity almost doubled at the wet site as compared to the dry site whereas moist conditions negatively affected fungal diversity and biomass (Fig. 1). In accordance, water saturation has unfavorable effect on soil fungal diversity and abundance in Arctic tundra soils [44, 98].

Fungal community composition on order and phylum levels differed substantially between the dry and the wet sites (Fig. 2). At the dry site, the majority of identified sequences belonged to *Ascomycota* with the most abundant order *Helotiales* reported as a dominant fungal group in Arctic soils [28, 30, 89]. Interestingly, *Basidiomycota* dominated the wet site (Fig. 2). With respect to functional groups, ectomycorrhizal fungi showed highest relative abundance at both sites, followed by lichenized fungi at the dry site and saprotrophic fungi at the wet site (Fig. 4), which is in accordance with a study from Alaskan tundra [41]. We conclude that ectomycorrhizal and saprotrophic fungi were more abundant at the wet site whereas conditions at the dry site favored lichenized fungi (Fig. 4a). However, taking fungal biomass at the individual sites into consideration, the dry site harbored a higher amount of ectomycorrhizal and saprotrophic fungi compared to the wet site (Fig. 4b), despite their lower sequence proportion. The higher proportion of lichenized fungi in dry tundra and almost no occurrence in wet tundra are likely due to denser vascular vegetation at the wet site, which outcompeted lichens preferring poorly vegetated habitats [25]. At the wet site ectomycorrhizal fungi distinctively dominated over saprotrophs likely due to more favorable soil conditions and increased shrub cover (e.g. of *Salix*) providing roots available for mycorrhizal colonization. Furthermore, mycorrhizal fungi can outcompete microbial decomposers for rhizosphere territory [53] or by decreasing nutrient availability in the soil [40], which may slow down SOM degradation and increase soil C storage [5]. Mycorrhizal fungi can also contribute to soil C storage by allocation of C from plants, as proposed by Clemmensen et al. [22]. However, it should be noted that comparisons of fungal functional groups in between the sites may be biased by the fact that the proportion of unidentified fungal taxa was higher at the dry site than at the wet site (Fig. 2).

Despite a large difference in bacterial diversity between the two sites (Fig. 1), bacterial community composition on phylum level showed rather minor differences between the dry and the wet sites (Fig. 2). *Proteobacteria*, *Actinobacteria* and *Acidobacteria* dominated at both sites in accordance with observations from Alaska and Svalbard [51, 52]. Nitrifying bacteria (ammonia-oxidizing and nitrite-oxidizing) significantly dominated at the wet site compared to the dry site (Additional file 7). This is in accordance with Alves et al. [2], who measured distinctively lower nitrification rates in dry soil compared to wet tundra soil. Contrary to nitrifying bacteria, nitrogen-fixing bacteria dominated at the dry site compared to the wet site. This was likely linked to a higher relative abundance of lichenized fungi at the dry site where nitrogen-fixing cyanobacteria form lichens, symbiotic relationship with lichenized fungi. Even though archaea represent only a minority of soil prokaryotes, in our study 0.6% in dry and 2.3% in wet tundra (Additional file 9), their role in greenhouse gas exchange by tundra soils is crucial. Ecosystems in the northern hemisphere with underlain permafrost soil represent the largest natural source of methane on Earth [37] and formation of methane (methanogenesis) is the terminal process of anaerobic C degradation performed solely by archaeal methanogens. Previous studies have shown consistent links between methanogen abundance and methanogenesis in Arctic soils [54, 88] and our observations indicate that soil moisture will likely be a key determinant of archaeal abundance and thus methane production.

### Direct and indirect effects of experimental enhancement of snow cover on microbial communities

Fungal and bacterial communities showed significant changes in response to short-term increased snow cover manipulation at both sites indicating high susceptibility and/or adaptability of Arctic soil microbes to climate change. Our findings are supported by previous studies on Arctic soils demonstrating fast increase in abundance of microbial functional genes involved in SOM decomposition [96] and enhanced litter decomposition rates [13, 20] as a result of short-term increase of snow pack. Interestingly, contrary to our hypothesis, the fungal community exhibited a stronger response to the manipulation compared to the bacteria (Fig. 5, Additional files 4, 5 and 8) even though bacteria are typically considered to have higher growth and turnover rates than fungi [80] thus possessing an ability to adapt faster to new conditions. To our knowledge, our study is the first to simultaneously analyze the response of fungal and bacterial communities in tundra soils to short-term climate change manipulations. However, our results find support by Semenova et al. [78] and Morgado et al. [58] who showed strong changes in fungal community composition as a response to elevated snow cover, and of Männistö et al. [57] who reported minor changes in bacterial communities under naturally elevated snow. It should be noted that the snow fence treatment has multiple direct (e.g. enhanced winter soil temperature, lowered soil temperature before and after snow melt, and elevated soil water content after snow melt) and indirect effects (e.g. shortened plant growing season due to late snow melt) on the microbial communities. It is difficult to disentangle the effect of these individual drivers on the microbial communities.

Deepened snow pack increased fungal biomass at the wet site and enhanced the diversity of fungi and bacteria at the dry site across all time points (Fig. 1). The effect on microbial diversity at the dry site was most pronounced at the beginning of the vegetative season, suggesting that higher and more stable temperatures during winter caused by increased snow pack (Additional file 2) may have a direct and positive effect on microbial metabolism and growth rates [16]. The increased snow pack likely prolonged the period in which a winter microbial community is able to build up. Higher abundance and diversity of microbes as a result of soil warming has been observed in different ecosystems [20, 69, 71, 96, 97] and increased microbial diversity is associated with the enhancement of various ecosystem processes including SOM decomposition [18, 68].

The most abundant fungal genera significantly affected by deepened snow were ectomycorrhizal *Inocybe, Camarophyllus, Russula, Tomentella,* and *Cenococcum* saprotrophic *Chalara*, *Mycena*, and *Clavaria*, saprotrophic yeast *Rhodotorula*, lichenized *Peltigera* and root-associated *Archaeorhizomyces* with uncertain ecological roles (Fig. 7). *Inocybe* represented the most abundant genus showing positive significant response to increased snow cover on both sites. Our observation contrasts Morgado et al. [58] and Semenova et al. [78] who observed a decline as well as Mundra et al. [61] showing no effect on abundance of *Inocybe* due to long-term snow manipulation. Ectomycorrhizal *Russula* and *Cenococcum* at the wet site and *Tomentella* at the dry site showed significantly higher abundance due to the treatment. Since fungi belonging to *Russula*, *Cenococcum* and *Tomentella* genera may possess decomposition abilities of complex soil organic matter [15, 26, 31], our observations might be the result of higher organic matter decomposition rates associated with increased soil temperature under elevated snow cover. The increased abundance of *Russula, Cenococcum* and *Tomentella* in response to soil warming has been reported in several studies [30, 35, 41, 65, 66]. Increased snow cover at the wet tundra site positively affected saprotrophic *Mycena* and *Clavaria.* Fungi belonging to *Mycena spp.*, which are able to degrade all the major components of plant litter [42], have been shown previously to increase as a response to soil warming [4, 81]. Our findings are supported by Blok et al. [13] who observed increased microbial litter decomposition in deep-snow plots (in the same experimental plots as used in our study) and agree with observations from Svalbard where an increase in saprotrophic fungi was observed after 6 year of snow fence manipulation [61]. A higher abundance of fungi with decomposing abilities in the treated plots indicates that the decomposition processes in tundra soils may change under future enhanced snow cover. Deepened snow pack significantly increased abundance of ectomycorrhizal fungi at the wet tundra site (Fig. 4b). Higher richness and abundance of ectomycorrhizal fungi as a response to summer soil warming has been reported previously [23, 29], but snow fence treatment has rather shown a decline in this community [58, 61, 78]. An increase in arbuscular mycorrhizal fungi due to deeper snow pack was detected in October at the wet tundra site (Fig. 4a). To our knowledge there are no previous reports of response of arbuscular mycorrhizal fungi to increased snow cover in tundra ecosystem. Contrary to our hypothesis, we did not detect any significant changes in abundance of saprotrophic fungi and plant pathogens under the snow cover manipulation which is in agreement with Semenova et al. [78] who did not observe any changes in abundance of these fungal functional groups after 18 years of increased snow depth in tundra soil.

Even though the response of prokaryotic community to increased snow cover was distinctively smaller than the response of fungi, several bacterial OTUs showed significant changes in their relative abundance under the treatment. The most abundant bacterial OTUs with significant feedback to snow manipulation were assigned to the classes *Acidobacteria*, *Actinobacteria*, *Sphingobacteria* and *Thermoleophilia* (Additional file 8). Representative taxa belonging to *Acidobacteria* and *Actinobacteria* have plant-specific interactions and abilities to degrade complex carbon compounds including plant cellulose and hemicellulose or fungal chitin [6, 34]. *Acidobacteria* and *Actinobacteria* are sensitive to environmental changes caused by increased snow cover [57, 73]. An abundant OTU assigned to *Chitinophagaceae* (*Sphingobacteriia* class), known as chitin degraders and hence able to decompose fungal cell wall material, responded positively to increased snow cover. In accordance, increased snow cover manipulation resulted in higher abundance of genes for enzymes involved in chitin utilization [73, 96]. Interestingly, the relative abundance of two abundant OTUs at the dry site assigned to thermo-tolerant cellulolytic *Acidothermaceae* decreased in response to the treatment. Our findings show changes in abundance of bacteria with polymer decomposing abilities, which indicates that increased snow cover can impact SOM decomposition processes driven not only by fungi, but also by bacteria. The archaeal community at the wet site showed decreased abundance due to snow fence treatment constantly across all sampling times, however, these changes were insignificant (Additional file 9). This contrasts Xue et al. [96] who reported higher archaeal abundance under snow fence treatment in a moist tundra.

## Conclusions

We demonstrate here that microbial communities in tundra soil differ at a small spatial scale due to contrasting soil parameters – in this case closely linked to landscape type, drainage and plant communities. Our results show that short-term climate manipulation (within few years) affects fungal and bacterial communities with fungal communities exhibiting a stronger response compared to prokaryotes. Rapid changes of fungal communities in response to the manipulation indicate their susceptibility and/or adaptability to current fluctuating weather conditions and future long-term climate changes. Furthermore, the effects of enhanced snow were manifested after snow had melted. Our study also shows that sampling at different time points within one growing season is needed if we are to understand the responses of soil microbial community composition to future changing climate. We are aware of the limitations of microbial rDNA amplicon sequencing [70], therefore for better understanding of the short-term effect of increased snow cover on microbial communities, it would be necessary to complement the current data with analysis of metatranscriptomes or metaproteomes providing insight into functional roles of individual microbial taxa under the future climate warming.

## Additional files

Additional file 1: Soil chemical properties. Data represent means and standard errors from four time points (*n* = 24). (PDF 11 kb)

Additional file 2: A) Daily mean temperatures in 5 cm soil depth in course of the year. The arrows indicate approximate soil sampling date. B) Monthly and yearly mean temperatures in degrees of Celsius in 5 cm soil depth in control and snow-manipulated dry and wet tundra sites. (PDF 315 kb)

Additional file 3: OTU accumulation curves expressed as the number of OTUs by number of reads from sequencing. (PDF 307 kb)

Additional file 4: Venn diagrams showing shifts in (a) fungal and (b) bacterial community composition. Proportion of shared and unique OTUs across four time points is displayed (*n* = 24). OTUs with > 0.01% abundance were considered as present. D-dry tundra, W-wet tundra, C-control, S-snow manipulation. (PDF 53 kb)

Additional file 5: Identification of the 30 most abundant fungal OTUs from dry and wet tundra soil according to UNITE database, their relative abundance in control (C) and snow manipulated plots (S) across the plant growing seasons (June, July, September and October) and seasonal average (SA). Data of relative abundance are expressed as means from 6 (24 for SA) replicates, standard errors are shown in italic. Statistically significant differences between control and snow manipulated plots in the individual seasons are highlighted (DESeq2, Benjamini-Hochberg correction, *p* < 0.05). Abundance (‰) represents mean relative abundance in all samples from particular tundra type. A- *Ascomycota*, B- *Basidiomycota*. (PDF 237 kb)

Additional file 6: Heatmap depicting the relative abundances of the 20 most dominant fungal genera in dry (a) and wet (b) tundra soil in control (C) and snow-manipulated (S) plots across the seasons. The data represent mean abundances (*n* = 6). Statistically significant effect (DESeq2, Benjamini-Hochberg correction, *P* < 0.05) between control (C) and snow-manipulated (S) plots in the individual seasons is indicated by asterisk. The color code relates to each OTU independently and indicates the relative abundance of each OTU where 100 (red) means the sample with the highest relative abundance for the specific OTU. (PDF 185 kb)

Additional file 7: Proportion of sequences belonging to different bacterial functional groups in dry (D) and wet (W) tundra soil in control (C) and snow-manipulated (S) plots. The data represent the means with standard errors (*n* = 6). (PDF 51 kb)

Additional file 8: Identification of the 30 most abundant bacterial OTUs from dry and wet tundra soil according to SILVA database, their relative abundance in control (C) and snow manipulated plots (S) across the plant growing seasons (June, July, September and October) and seasonal average (SA). Data of relative abundance are expressed as means from 6 (24 for SA) replicates, standard errors are shown in italic. Statistically significant differences between control and snow manipulated plots in the individual seasons are highlighted (DESeq2, Benjamini-Hochberg correction, *p* < 0.05). Abundance (‰) represents mean relative abundance in all samples from particular tundra type. (PDF 147 kb)

Additional file 9: Relative abundance of archaea in prokaryotic sequence pool in dry (A) and wet (B) tundra soil in control (green) and snow-manipulated (red) plots by season and as a seasonal average (SA). The data represent the means with standard errors (*n* = 6), for seasonal average (*n* = 24). (PDF 57 kb)

## Data Availability

Sequencing data are available in MGRAST public database (http://metagenomics.anl.gov/, dataset numbers 4782666.3 and 4782667.3).

## Abbreviations

ANOSIM: Analysis of similarity
C: Carbon
N: Nitrogen
OTU: Operational taxonomic unit
PCA: Principal component analysis
PCR: Polymerase chain reaction
RDA: Redundancy analysis
SOC: Soil organic carbon
SOM: Soil organic matter
