# Supplementary material for: Fast response of fungal and prokaryotic communities to climate change manipulation in two contrasting tundra soils
Source: Environ Microbiome. 2019 Sep 18;14:6. doi: 10.1186/s40793-019-0344-4 (PMC7989089; doi:10.1186/s40793-019-0344-4)
Supplement: Supplementary file 1 — Soil chemical properties. Data represent means and standard errors from four time points (n = 24). (PDF 11 kb) [file 40793_2019_344_MOESM1_ESM.pdf]

Additional file 1

Soil chemical properties. Data represent means and standard errors from four time points (n=24).

	DC	DS	WC	WS
C (%)	19.4 ± 1.38	20.7 ± 1.54	26.2 ± 1.36	27.4 ± 1.70
N (%)	0.58 ± 0.03	0.61 ± 0.04	1.46 ± 0.12	1.46 ± 0.13
C/N ratio	33.9 ± 1.81	34.1 ± 1.84	21.3 ± 2.21	22.4 ± 2.31
water content (%)	52.2 ± 2.72	53.8 ± 2.66	80.2 ± 1.35	79.7 ± 1.78
pH	5.16 ± 0.05	5.14 ± 0.07	5.82 ± 0.03	5.89 ± 0.03
organic matter (%)	38.1 ± 2.27	40.4 ± 2.78	55.8 ± 2.47	58.7 ± 3.02
