# Supplementary material for: Fast response of fungal and prokaryotic communities to climate change manipulation in two contrasting tundra soils
Source: Environ Microbiome. 2019 Sep 18;14:6. doi: 10.1186/s40793-019-0344-4 (PMC7989089; doi:10.1186/s40793-019-0344-4)
Supplement: Supplementary file 2 — A) Daily mean temperatures in 5 cm soil depth in course of the year. The arrows indicate approximate soil sampling date. B) Monthly and yearly mean temperatures in degrees of Celsius in 5 cm soil depth in control and snow-manipulated dry and wet tundra sites. (PDF 315 kb) [file 40793_2019_344_MOESM2_ESM.pdf]

Additional file 2

A) Daily mean temperatures in 5 cm soil depth in course of the year. The arrows indicate approximate soil sampling date.

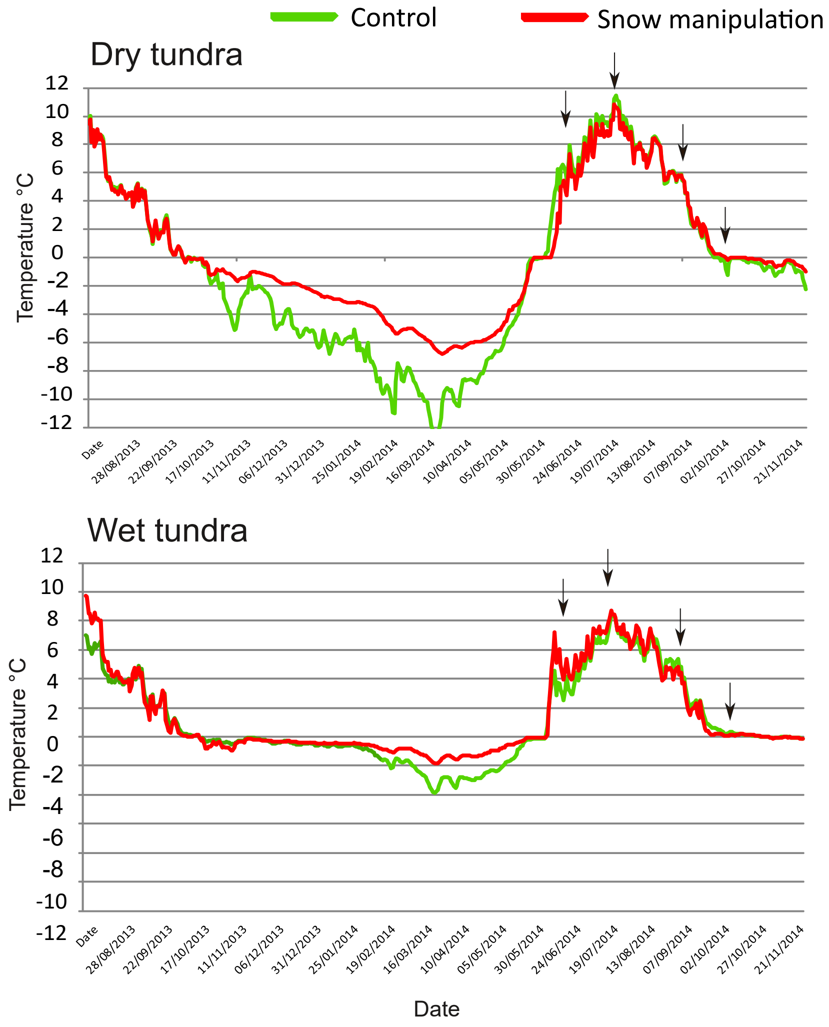

B) Monthly and yearly mean temperatures in degrees of Celsius in 5 cm soil depth in control and snow-manipulated dry and wet tundra sites.

	October 13	November 13	December 13	January 14	February 14	March 14	April 14	May 14	June 14	July 14	August 14	September 14	yearly average
Dry Control	-0.46	-2.88	-4.43	-5.81	-8.19	-9.82	-8.84	-4.25	3.94	9.35	7.55	7.84	-1.33
Dry Snow	-0.29	-1.21	-1.77	-2.87	-4.01	-5.69	-6.14	-3.47	2.84	8.67	7.39	6.82	0.02
Wet Control	0.18	-0.26	-0.39	-0.91	-1.24	-2.48	-2.90	-1.50	2.12	6.44	6.13	2.91	0.68
Wet Snow	0.00	-0.43	-0.32	-1.27	-0.71	-1.20	-1.35	-0.59	3.00	6.90	6.21	2.41	1.06
