# Supplementary material for: Fast response of fungal and prokaryotic communities to climate change manipulation in two contrasting tundra soils
Source: Environ Microbiome. 2019 Sep 18;14:6. doi: 10.1186/s40793-019-0344-4 (PMC7989089; doi:10.1186/s40793-019-0344-4)

Additional file 3

OTU accumulation curves expressed as the number of OTUs by number of reads from sequencing. D-dry tundra, W-wet tundra, C-control, S-snow manipulation.

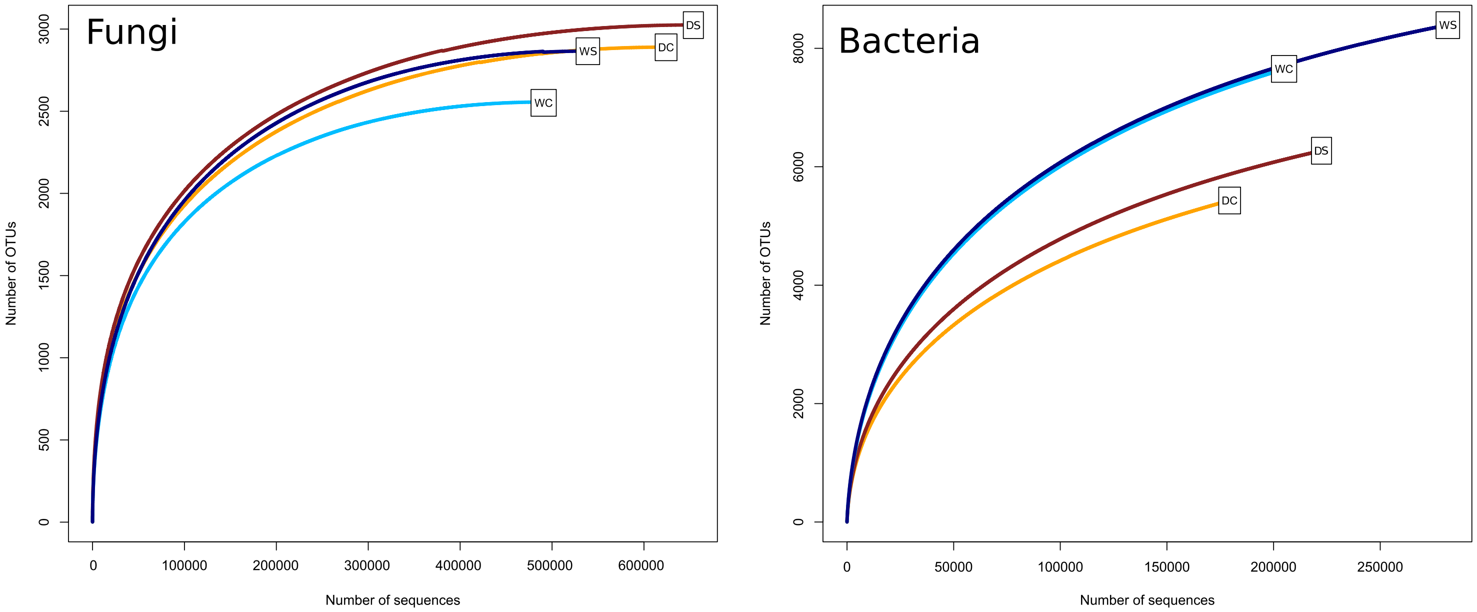

Supplement: Supplementary file 3 — OTU accumulation curves expressed as the number of OTUs by number of reads from sequencing. (PDF 307 kb) [file 40793_2019_344_MOESM3_ESM.pdf]
