# Supplementary material for: Fast response of fungal and prokaryotic communities to climate change manipulation in two contrasting tundra soils
Source: Environ Microbiome. 2019 Sep 18;14:6. doi: 10.1186/s40793-019-0344-4 (PMC7989089; doi:10.1186/s40793-019-0344-4)

Additional file 4

Venn diagrams showing shifts in (a) fungal and (b) bacterial community composition. Proportion of shared and unique OTUs across four time points is displayed (n=24). OTUs with > 0.01% abundance were considered as present. D-dry tundra, W-wet tundra, C-control, S-snow manipulation.

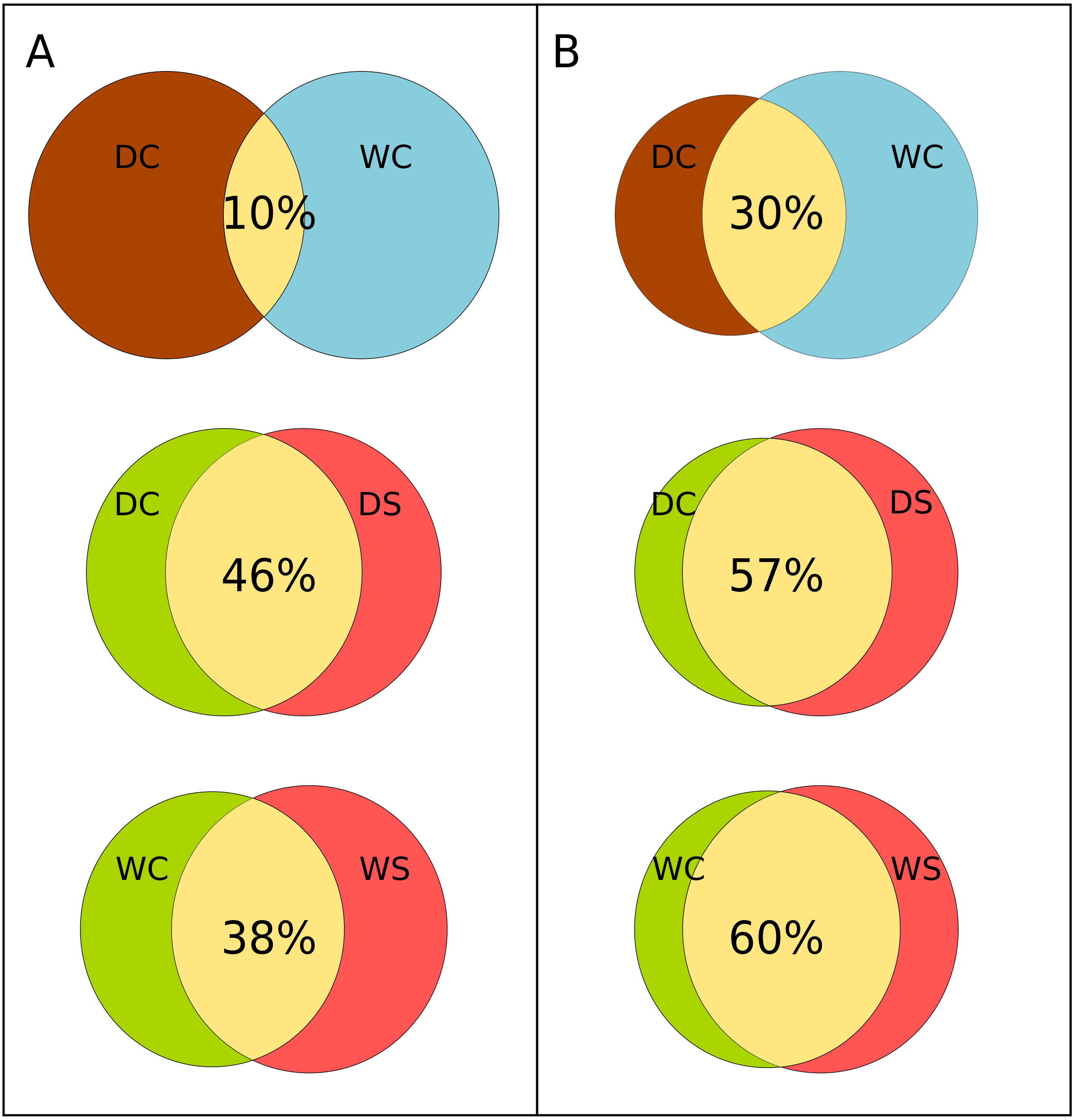

Supplement: Supplementary file 4 — Venn diagrams showing shifts in (a) fungal and (b) bacterial community composition. Proportion of shared and unique OTUs across four time points is displayed (n = 24). OTUs with > 0.01% abundance were considered as present. D-dry tundra, W-wet tundra, C-control, S-snow manipulation. (PDF 53 kb) [file 40793_2019_344_MOESM4_ESM.pdf]
