# Supplementary material for: Fast response of fungal and prokaryotic communities to climate change manipulation in two contrasting tundra soils
Source: Environ Microbiome. 2019 Sep 18;14:6. doi: 10.1186/s40793-019-0344-4 (PMC7989089; doi:10.1186/s40793-019-0344-4)
Supplement: Supplementary file 5 — Identification of the 30 most abundant fungal OTUs from dry and wet tundra soil according to UNITE database, their relative abundance in control (C) and snow manipulated plots (S) across the plant growing seasons (June, July, September and October) and seasonal average (SA). Data of relative abundance are expressed as means from 6 (24 for SA) replicates, standard errors are shown in italic. Statistically significant differences between control and snow manipulated plots in the individual seasons are highlighted (DESeq2, Benjamini-Hochberg correction, p < 0.05). Abundance (‰) represents mean relative abundance in all samples from particular tundra type. A- Ascomycota, B- Basidiomycota. (PDF 237 kb) [file 40793_2019_344_MOESM5_ESM.pdf]

Additional file 5

Identification of the 30 most abundant fungal OTUs from dry and wet tundra soil according to UNITE database, their relative abundance in control (C) and snow manipulated plots (S) across the plant growing seasons (June, July, September and October) and seasonal average (SA). Data of relative abundance are expressed as means from 6 (24 for SA) replicates, standard errors are shown in italic. Statistically significant differences between control and snow manipulated plots in individual seasons are highlighted (DESeq2, Benjamini-Hochberg correction, $p<0.05$). Abundance (‰) represents mean relative abundance in all samples from particular tundra type. Fungal divisions: A - *Ascomycota*, B – *Basidiomycota*.

DRY TUNDRA

						Relative abundance(‰)									
OTU	Order(division)	Best identified hit (accession number)	Similarity (%)	E-value	Abundance (‰)	June C	June S	July C	July S	Sept C	Sept S	Oct C	Oct S	SA C	SA S
1	(A)	*Lecanoromycetes* sp. (FJ554378)	100	7E-68	51.1	73.5	32.9	36.1	50.4	42.0	53.0	62.3	58.4	53.5	48.7
						9.5	*10.5*	*3.4*	*12.1*	*8.5*	*14.3*	*9.6*	*15.5*	*4.8*	*6.4*
2	*Helotiales* (A)	uncultured *Helotiales* (HQ211989)	100	2E-69	44.3	45.7	31.6	50.8	36.2	50.7	50.4	52.9	35.9	50.0	38.5
						9.6	*4.5*	*7.5*	*5.8*	*8.2*	*11.3*	*9.9*	*10.8*	*4.1*	*4.4*
5	*Chaetothyriales* (A)	*Capronia* sp. (GQ160174)	94	8E-58	37.4	39.0	39.8	37.4	41.4	25.9	19.7	50.1	45.6	38.1	36.6
						17.4	*10.2*	*13.3*	*12.8*	*5.7*	*7.9*	*16.3*	*11.5*	*6.6*	*5.4*
6	(A)	*Dothideomycetes* sp. (HQ445490)	99	1E-70	29.1	18.2	30.9	22.8	34.5	23.7	47.3	28.8	26.8	23.4	34.9
						7.3	*9.5*	*7.0*	*11.9*	*4.8*	*17.7*	*10.4*	*8.9*	*3.6*	*6.0*
11	*Chaetothyriales* (A)	*Capronia* sp. (GQ160174)	94	8E-58	25.2	19.3	28.0	17.2	14.5	21.1	21.8	52.4	27.0	27.5	22.8
						11.4	*12.0*	*6.6*	*8.7*	*9.0*	*9.2*	*16.4*	*19.1*	*6.0*	*6.2*
13	unidentified	uncultured fungus (KF617375)	100	8E-78	22.5	25.1	16.7	26.3	18.2	19.7	18.5	28.9	27.0	25.0	20.1
						11.9	*4.3*	*7.5*	*6.1*	*3.8*	*3.6*	*13.2*	*10.0*	*4.6*	*3.1*
9	unidentified	uncultured fungus (KF617966)	100	1E-76	22.1	12.4	21.0	26.8	22.9	15.4	21.1	21.4	35.9	19.0	25.2
						3.6	*6.5*	*10.2*	*9.1*	*2.7*	*8.5*	*8.5*	*10.3*	*3.4*	*4.3*
16	(A)	*Lecanoromycetes* sp. (HQ445264)	100	1E-71	14.1	12.6	11.3	16.6	13.3	17.6	11.0	20.5	10.3	**16.8**	**11.5**
						1.9	*2.3*	*5.0*	*2.9*	*3.3*	*2.2*	*5.8*	*2.5*	*2.0*	*1.2*
22	*Archaeorhizomycetales* (A)	*Archaeorhizomyces* sp. (GU174299)	91	9E-32	14.0	19.6	19.3	12.0	11.3	14.3	11.8	12.0	11.8	14.5	13.6
						7.0	*4.5*	*4.3*	*3.6*	*4.2*	*2.5*	*4.9*	*4.3*	*2.5*	*1.9*
21	*Helotiales* (A)	*Helotiales* sp. (HM230877)	90	7E-38	13.1	12.4	5.6	14.4	10.7	27.7	8.3	19.3	6.7	**18.5**	**7.8**
						3.1	*2.2*	*2.9*	*3.9*	*17.2*	*2.9*	*4.3*	*3.7*	*4.3*	*1.6*
17	unidentified	uncultured fungus (JN889821)	100	3E-56	13.1	1.8	28.2	5.4	9.8	1.1	35.6	6.2	16.5	3.6	22.5
						1.2	*18.2*	*2.2*	*6.0*	*0.5*	*24.4*	*5.1*	*10.3*	*1.4*	*7.9*
32	*Agaricales* (B)	*Camarophyllus* sp. (GU233366)	88	1E-27	12.9	49.9	0.2	50.3	0.0	0.1	1.5	0.4	0.8	**25.2**	**0.6**
						49.9	*0.1*	*31.8*	*0.0*	*0.1*	*1.5*	*0.4*	*0.8*	*14.5*	*0.4*
20	*Agaricales* (B)	*Clavaria* sp. (EF434000)	98	5E-101	12.6	5.8	13.3	8.3	25.1	10.0	6.2	11.3	20.4	8.9	16.2
						1.4	*4.4*	*2.3*	*17.5*	*2.9*	*1.6*	*2.3*	*11.7*	*1.1*	*5.1*
27	(A)	*Leotiomycetes* sp. (HQ211917)	100	3E-71	12.5	9.8	13.8	12.1	13.8	19.0	14.8	8.2	8.9	12.3	12.8
						4.6	*3.4*	*2.8*	*4.7*	*8.0*	*4.3*	*2.1*	*2.7*	*2.4*	*1.8*
26	unidentified	uncultured fungus (KF617433)	100	4E-81	12.4	16.2	10.2	19.9	6.5	11.7	5.8	17.7	11.5	**16.4**	**8.5**
						5.1	*5.1*	*6.5*	*2.4*	*3.2*	*1.6*	*5.3*	*4.3*	*2.5*	*1.7*
24	*Agaricales* (B)	*Inocybe leiocephala* (AM882793)	100	2E-115	11.9	**0.2**	**29.8**	**0.3**	**13.5**	0.2	21.8	**1.5**	**27.7**	**0.5**	**23.2**
						0.1	*12.5*	*0.2*	*9.4*	*0.1*	*13.6*	*1.4*	*14.5*	*0.3*	*6.0*
30	unidentified	uncultured fungus (KC965680)	92	8E-53	11.8	20.7	9.4	16.4	8.8	9.3	7.3	14.9	8.0	15.3	8.3
						12.8	*3.2*	*3.8*	*2.9*	*2.4*	*1.6*	*5.8*	*2.5*	*3.5*	*1.2*
28	*Archaeorhizomycetales* (A)	*Archaeorhizomyces* sp. (JN889847)	100	6E-58	11.6	9.6	11.3	8.8	9.3	19.4	20.6	9.5	4.0	11.8	11.3
						3.2	*2.1*	*3.2*	*3.1*	*6.0*	*6.4*	*4.4*	*1.4*	*2.5*	*2.1*
33	*Helotiales* (A)	*Helotiales* sp. (AM260904)	90	5E-45	10.4	6.3	6.6	12.4	9.3	26.4	8.2	7.4	6.8	13.1	7.7
						2.9	*1.7*	*3.5*	*2.9*	*14.5*	*2.5*	*2.3*	*2.0*	*3.9*	*1.1*
29	*Pleosporales* (A)	*Venturia* sp. (HQ211781)	99	2E-69	9.8	3.8	18.2	10.4	16.3	6.6	4.1	8.7	10.1	7.4	12.2

						1.6	*9.0*	*4.6*	*8.0*	*2.4*	*1.8*	*3.0*	*2.8*	*1.5*	*3.1*
50	*Agaricales* (B)	*Cuphophyllus lacmus* (JQ272404)	96	5E-91	9.4	3.7	2.3	8.9	13.1	19.4	18.6	2.0	6.9	8.5	10.2
						3.6	*2.3*	*7.8*	*12.0*	*10.0*	*12.8*	*2.0*	*6.8*	*3.6*	*4.5*
45	*Boletales* (B)	*Leccinum rotundifoliae* (GU234155)	100	3E-154	9.4	7.7	24.1	9.9	7.2	10.0	10.9	3.3	1.8	7.7	11.0
						5.4	*17.6*	*7.4*	*5.9*	*5.3*	*10.2*	*1.2*	*1.2*	*2.5*	*5.1*
41	unidentified	uncultured fungus (KC965680)	92	8E-53	9.3	3.2	8.9	7.6	7.6	5.5	24.0	9.3	8.1	6.4	12.2
						2.2	*7.2*	*5.8*	*4.5*	*4.6*	*13.3*	*5.9*	*5.8*	*2.4*	*4.1*
43	*Agaricales* (B)	*Cortinariaceae* sp. (HE979020)	100	2E-94	9.1	3.7	6.8	0.2	16.8	12.3	20.2	2.0	11.1	4.6	13.7
						3.7	*5.9*	*0.1*	*13.3*	*12.3*	*13.2*	*2.0*	*8.8*	*3.1*	*5.1*
36	*Mortierellales*	*Mortierellales* sp. (KM113749)	100	1E-122	9.1	8.0	16.3	8.8	11.0	4.7	8.1	4.4	11.8	6.5	11.8
						2.5	*7.1*	*1.9*	*4.6*	*0.5*	*2.6*	*0.9*	*2.8*	*0.9*	*2.2*
44	*Chaetothyriales* (A)	*Capronia* sp. (GQ160174)	92	4E-46	8.8	7.8	8.8	9.2	8.9	6.0	12.4	10.6	6.5	8.4	9.2
						3.5	*5.4*	*2.9*	*3.7*	*2.4*	*3.9*	*3.6*	*2.5*	*1.5*	*1.9*
49	unidentified	uncultured fungus (KF617857)	100	1E-80	8.4	11.4	8.0	9.2	3.6	8.6	6.7	12.3	7.6	**10.4**	**6.5**
						1.3	*2.9*	*1.4*	*1.3*	*1.4*	*3.1*	*5.6*	*2.6*	*1.4*	*1.2*
48	(A)	*Lecanoromycetes* sp. (KC965673)	99	2E-64	8.0	9.7	12.5	**3.6**	**11.9**	4.7	4.2	6.3	10.7	6.1	9.8
						5.1	*3.1*	*0.6*	*2.9*	*1.5*	*1.4*	*1.3*	*2.4*	*1.3*	*1.4*
56	unidentified	uncultured fungus (KC965988)	98	2E-100	7.2	9.0	4.9	6.2	5.5	6.7	8.7	7.9	9.1	7.4	7.1
						4.1	*2.0*	*1.6*	*1.7*	*3.1*	*5.6*	*3.8*	*3.6*	*1.5*	*2.1*
40	*Helotiales* (A)	*Phialocephala* sp. (KJ817277)	100	3E-71	7.2	8.0	5.6	11.6	5.4	5.9	7.8	6.0	7.4	7.9	6.6
						2.2	*1.1*	*2.5*	*0.8*	*1.2*	*1.3*	*1.4*	*1.7*	*1.0*	*0.6*

WET TUNDRA

						Relative abundance(‰)									
OTU	Order(division)	Best identified hit (accession number)	Similarity (%)	E-value	Abundance (‰)	June C	June S	July C	July S	Sept C	Sept S	Octo C	Octo S	SA C	SA S
0	*Agaricales*(B)	*Mycenella* sp. (JF519051)	93	5E-76	92.5	97.6	72.4	132.7	128.5	76.0	60.1	**146.9**	**25.7**	113.3	71.7
						24.7	*23.5*	*49.0*	*39.7*	*25.4*	*28.0*	*53.0*	*9.5*	*19.6*	*14.9*
3	*Agaricales*(B)	*Inocybe* sp. (HQ215790)	100	8E-94	50.4	24.7	90.1	19.0	29.4	**12.5**	**90.4**	**13.0**	**124.2**	**17.3**	**83.5**
						10.5	*28.3*	*10.4*	*8.6*	*8.9*	*24.1*	*6.1*	*39.1*	*4.4*	*14.6*
4	*Agaricales* (B)	*Inocybe bulbosissima* (JX436913)	100	1E-91	45.3	43.7	79.4	27.2	22.9	66.2	40.6	20.7	61.6	39.5	51.1
						23.3	*41.5*	*18.0*	*12.2*	*24.3*	*18.4*	*6.8*	*20.7*	*9.7*	*12.8*
8	*Agaricales* (B)	*Cortinarius* sp. (JX630756)	99	2E-89	34.6	34.4	29.2	57.1	45.6	46.7	11.2	35.6	17.0	43.4	25.8
						19.7	*6.4*	*20.6*	*17.6*	*15.1*	*4.8*	*8.8*	*4.3*	*8.0*	*5.4*
10	*Agaricales* (B)	*Inocybaceae* sp. (KC965475)	100	5E-101	29.8	43.9	16.8	24.8	10.6	30.4	28.6	43.0	40.6	35.5	24.1
						21.5	*2.0*	*7.5*	*3.5*	*9.9*	*9.4*	*8.5*	*10.7*	*6.4*	*4.2*
7	unidentified	uncultured fungus (JX135682)	99	4E-71	28.9	28.0	21.2	2.4	14.5	15.0	3.0	51.4	96.0	24.2	33.7
						26.2	*12.1*	*1.0*	*7.1*	*10.4*	*2.0*	*34.7*	*51.7*	*11.1*	*14.6*
12	*Pezizales* (A)	*Geopora nicaeensis* (JF908022)	100	2E-83	26.8	25.8	20.2	17.0	27.3	32.5	33.2	24.6	33.8	25.0	28.6
						4.6	*6.6*	*5.0*	*8.1*	*11.2*	*13.2*	*6.0*	*8.1*	*3.6*	*4.5*
14	*Agaricales* (B)	uncultured *Cortinarius* (JX630423)	99	3E-88	22.3	36.9	11.6	42.2	34.1	21.5	6.7	13.2	12.3	28.4	16.2
						29.9	*4.4*	*24.6*	*13.4*	*10.8*	*2.2*	*4.9*	*4.8*	*9.8*	*4.1*
15	*Agaricales* (B)	uncultured *Cortinarius* (KM403065)	100	8E-94	18.2	5.4	25.8	3.1	53.9	18.1	8.3	20.2	10.5	11.7	24.6
						5.2	*25.3*	*2.5*	*47.4*	*16.5*	*4.0*	*5.0*	*6.5*	*4.5*	*13.2*
23	*Russulales* (B)	*Russula brevipes* (JX630807)	100	3E-129	17.8	0.0	20.8	0.0	7.8	14.2	49.0	**0.0**	**50.6**	**3.6**	**32.1**
						0.0	*17.7*	*0.0*	*7.7*	*14.2*	*49.0*	*0.0*	*32.5*	*3.6*	*14.9*
19	*Agaricales* (B)	*Inocybe fraudans* (AM882732)	100	1E-107	17.2	0.3	23.9	45.2	4.6	25.5	18.8	2.7	16.7	18.4	16.0
						0.2	*20.9*	*42.5*	*3.8*	*25.5*	*18.8*	*2.1*	*14.4*	*12.2*	*7.5*
18	*Agaricales* (B)	uncultured *Inocybe* (KJ792557)	100	5E-101	16.3	34.4	15.2	2.8	9.0	10.5	3.9	27.9	26.7	18.9	13.7
						15.9	*11.3*	*2.0*	*6.5*	*5.2*	*1.6*	*17.2*	*7.7*	*6.2*	*4.0*
31	*Thelephorales* (B)	uncultured *Tomentella* (KC455330)	100	1E-111	13.7	12.4	9.3	14.1	7.8	13.5	12.4	22.1	17.8	15.5	11.8
						5.2	*2.9*	*4.7*	*1.6*	*9.3*	*5.3*	*7.0*	*8.3*	*3.3*	*2.6*
25	*Thelephorales* (B)	*Thelephoraceae* sp. (UDB008805)	98	5E-106	13.3	17.0	28.3	5.9	14.6	3.6	11.4	15.5	10.0	10.5	16.1
						8.3	*10.0*	*4.4*	*6.5*	*2.1*	*6.7*	*7.9*	*4.9*	*3.2*	*3.7*
34	unidentified (A)	*Leotiomycetes* sp. (KF618056)	96	3E-61	12.3	8.5	6.3	23.9	25.6	11.4	11.4	6.8	4.5	12.6	11.9
						2.9	*2.3*	*8.0*	*12.2*	*4.4*	*5.7*	*1.9*	*0.7*	*2.7*	*3.6*
38	*Pleosporales* (A)	*Lindgomyces apiculatus*(JQ435794)	97	3E-67	11.7	9.5	8.4	25.5	4.7	20.5	10.0	9.8	5.4	**16.3**	**7.2**

						2.4	*2.5*	*7.3*	*1.0*	*5.4*	*4.3*	*3.9*	*1.7*	*2.8*	*1.3*
39	*Agaricales* (B)	*Inocybe aff. hystrix*(GU949589)	90	4E-32	11.5	11.6	4.0	0.2	7.9	3.4	37.7	**24.6**	**2.6**	10.0	13.1
						11.5	*2.6*	*0.1*	*4.7*	*2.4*	*21.3*	*12.6*	*1.3*	*4.5*	*5.9*
42	*Thelephorales* (B)	*Tomentella* sp. (EU645643)	100	3E-113	10.9	3.3	8.3	7.3	11.6	9.6	11.5	25.6	9.7	11.5	10.3
						0.9	*1.6*	*4.9*	*3.1*	*4.8*	*5.4*	*7.5*	*4.8*	*3.0*	*1.9*
52	*Agaricales* (B)	*Inocybe polytrichi-norvegici* (JF908185)	94	1E-86	10.5	13.3	3.1	37.4	1.0	8.8	0.0	**20.0**	**0.3**	19.9	1.1
						7.6	*3.0*	*16.8*	*0.9*	*6.2*	*0.0*	*10.1*	*0.1*	*5.6*	*0.8*
46	unidentified	uncultured fungus (KC966138)	100	1E-71	10.4	20.0	8.3	10.6	8.1	19.0	5.3	8.0	4.2	14.4	6.5
						5.6	*2.2*	*3.3*	*2.5*	*6.0*	*1.5*	*2.9*	*1.4*	*2.4*	*1.0*
51	*Agaricales* (B)	uncultured *Laccaria* (KJ792593)	100	6E-105	10.2	5.2	9.5	26.7	3.3	10.7	6.8	10.2	9.3	13.2	7.2
						1.9	*6.7*	*23.7*	*1.3*	*5.2*	*3.0*	*3.9*	*3.6*	*6.0*	*2.0*
47	*Sebacinales* (B)	uncultured *Sebacinales* (FJ553298)	100	1E-102	9.7	25.5	3.9	8.8	4.1	17.2	2.0	11.6	4.4	**15.8**	**3.6**
						9.2	*1.2*	*2.7*	*1.0*	*6.8*	*0.6*	*3.7*	*1.0*	*3.2*	*0.5*
37	unidentified (A)	*Dothideomycetes* sp. (JQ759636)	100	3E-72	9.1	7.6	3.9	17.2	12.1	4.6	5.9	15.1	6.3	10.9	7.5
						3.2	*1.1*	*13.5*	*6.8*	*2.3*	*3.3*	*8.7*	*1.7*	*1.7*	*1.3*
62	*Agaricales* (B)	uncultured *Inocybe* (KJ792586)	100	8E-99	7.7	20.7	0.1	4.3	0.8	**20.6**	**0.0**	**15.1**	**0.2**	**11.1**	**7.0**
						20.0	*0.1*	*4.0*	*0.8*	*12.0*	*0.0*	*12.6*	*0.1*	*4.0*	*1.9*
58	*Sebacinales* (B)	uncultured *Tremellodendron* (KJ792733)	100	2E-100	7.1	3.9	2.3	3.2	10.5	29.7	2.7	3.0	1.5	15.2	0.3
						3.8	*2.2*	*2.9*	*10.2*	*26.9*	*2.0*	*1.9*	*1.0*	*6.4*	*0.2*
66	*Thelephorales* (B)	uncultured *Thelephoraceae* (KF514676)	98	3E-103	6.6	11.1	9.6	1.6	1.5	2.3	5.4	17.5	4.1	10.0	4.3
						7.2	*4.7*	*0.7*	*0.4*	*1.0*	*1.9*	*8.9*	*0.8*	*6.8*	*2.6*
67	*Agaricales* (B)	*Hemimycena pseudolactea* (UDB017907)	93	2E-69	6.6	5.0	6.1	0.6	3.7	9.5	25.2	1.3	1.3	8.1	5.1
						3.6	*2.9*	*0.2*	*2.1*	*2.4*	*17.4*	*0.6*	*0.5*	*3.0*	*1.3*
82	unidentified (A)	*Leotiomycetes* sp. (KF297145)	95	3E-56	6.1	5.1	5.7	10.6	4.8	7.8	1.6	8.5	4.7	4.1	9.1
						1.9	*2.0*	*6.3*	*2.3*	*4.2*	*0.9*	*2.1*	*2.0*	*1.3*	*4.6*
77	unidentified	uncultured fungus (KC965555)	97	1E-61	6.0	10.1	5.1	17.2	4.9	8.0	1.7	0.5	0.6	8.0	4.2
						5.9	*2.5*	*10.4*	*2.4*	*6.8*	*1.0*	*0.2*	*0.2*	*1.9*	*0.9*
64	*Thelephorales (B)*	*Tomentella* sp. (FN687658)	99	3E-108	5.8	0.3	5.3	0.0	7.2	1.2	6.3	**0.0**	**25.7**	**1.5**	**11.1**
						0.3	*4.0*	*0.0*	*6.2*	*1.2*	*5.5*	*0.0*	*15.2*	*0.8*	*4.0*
