# Supplementary material for: Fast response of fungal and prokaryotic communities to climate change manipulation in two contrasting tundra soils
Source: Environ Microbiome. 2019 Sep 18;14:6. doi: 10.1186/s40793-019-0344-4 (PMC7989089; doi:10.1186/s40793-019-0344-4)

Additional file 6

Heatmap depicting the relative abundances of 20 most dominant fungal genera in dry (a) and wet (b) tundra soil in control (C) and snow-manipulated (S) plots across the seasons. The data represent mean abundances (n=6). Statistically significant effect (DESeq2, Benjamini-Hochberg correction, P<0.05) between control (C) and snow-manipulated (S) plots in individual seasons is indicated by asterisk. The color code relates to each OTU independently and indicates the relative abundance of each OTU where 100 (red) means the sample with the highest relative abundance for the specific OTU.

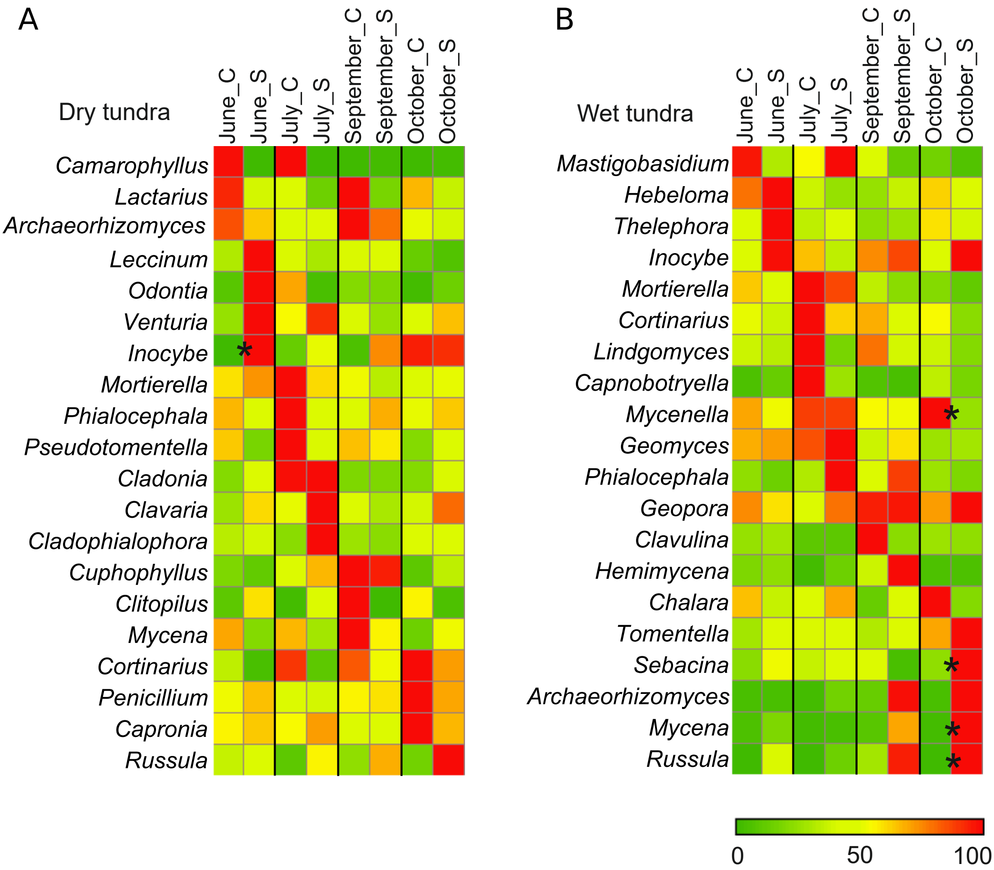

Supplement: Supplementary file 6 — Heatmap depicting the relative abundances of the 20 most dominant fungal genera in dry (a) and wet (b) tundra soil in control (C) and snow-manipulated (S) plots across the seasons. The data represent mean abundances (n = 6). Statistically significant effect (DESeq2, Benjamini-Hochberg correction, P < 0.05) between control (C) and snow-manipulated (S) plots in the individual seasons is indicated by asterisk. The color code relates to each OTU independently and indicates the relative abundance of each OTU where 100 (red) means the sample with the highest relative abundance for the specific OTU. (PDF 185 kb) [file 40793_2019_344_MOESM6_ESM.pdf]
