# Supplementary material for: Fast response of fungal and prokaryotic communities to climate change manipulation in two contrasting tundra soils
Source: Environ Microbiome. 2019 Sep 18;14:6. doi: 10.1186/s40793-019-0344-4 (PMC7989089; doi:10.1186/s40793-019-0344-4)

Additional file 7

Proportion of sequences belongning to different bacterial functional groups in dry (D) and wet (W) tundra soil in control (C) and snow-manipulated (S) plots. The data represent the means with standard errors (n=6).

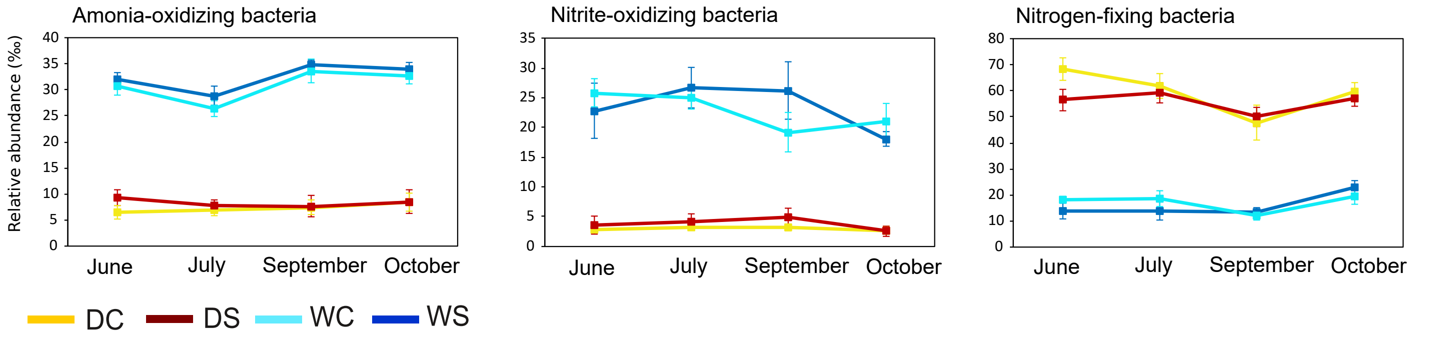

Supplement: Supplementary file 7 — Proportion of sequences belonging to different bacterial functional groups in dry (D) and wet (W) tundra soil in control (C) and snow-manipulated (S) plots. The data represent the means with standard errors (n = 6). (PDF 51 kb) [file 40793_2019_344_MOESM7_ESM.pdf]
