# Supplementary material for: Fast response of fungal and prokaryotic communities to climate change manipulation in two contrasting tundra soils
Source: Environ Microbiome. 2019 Sep 18;14:6. doi: 10.1186/s40793-019-0344-4 (PMC7989089; doi:10.1186/s40793-019-0344-4)
Supplement: Supplementary file 8 — Identification of the 30 most abundant bacterial OTUs from dry and wet tundra soil according to SILVA database, their relative abundance in control (C) and snow manipulated plots (S) across the plant growing seasons (June, July, September and October) and seasonal average (SA). Data of relative abundance are expressed as means from 6 (24 for SA) replicates, standard errors are shown in italic. Statistically significant differences between control and snow manipulated plots in the individual seasons are highlighted (DESeq2, Benjamini-Hochberg correction, p < 0.05). Abundance (‰) represents mean relative abundance in all samples from particular tundra type. (PDF 147 kb) [file 40793_2019_344_MOESM8_ESM.pdf]

Additional file 8

Identification of the 30 most abundant bacterial OTUs from dry and wet tundra soil according to SILVA database, their relative abundance in control (C) and snow manipulated plots (S) across the plant growing seasons (June, July, September and October) and seasonal average (SA). Data of relative abundance are expressed as means from 6 (24 for SA) replicates, standard errors are shown in italic. Statistically significant differences between control and snow manipulated plots in individual seasons are highlighted (DESeq2, Benjamini-Hochberg correction, $p<0.05$). Abundance (‰) represents mean relative abundance in all samples from particular tundra type.

DRY TUNDRA

						Relative abundance (‰)									
OTU	Class	Best identified hit (accession number)	Similarity (%)	E-value	Abundance (‰)	June C	June S	July C	July S	Sept C	Sept S	Oct C	Oct S	SA C	SA S
3	*Alphaproteobacteria*	*Bradyrhizobium* sp. (JQ357060)	100	9E-130	52.8	63.8	51.5	57.0	55.4	43.9	45.3	53.9	51.7	54.6	51.0
						2.8	*3.4*	*3.9*	*3.5*	*6.2*	*3.4*	*3.2*	*3.5*	*2.5*	*1.7*
1	*Spartobacteria*	*Chthoniobacterales* sp. (JQ368031)	100	9E-130	36.8	30.0	36.8	37.4	42.9	41.4	37.9	33.5	34.2	35.6	38.0
						4.5	*6.3*	*4.8*	*7.5*	*6.3*	*8.3*	*6.6*	*7.9*	*2.7*	*3.5*
9	*Thermoleophilia*	*Solirubrobacterales* sp. (JQ369408)	100	9E-130	29.9	34.6	30.2	37.1	33.0	24.7	27.6	26.4	25.3	30.7	29.0
						3.4	*4.5*	*3.8*	*4.7*	*3.2*	*4.6*	*3.2*	*4.8*	*1.9*	*2.2*
11	*Acidobacteria*	*Acidobacteria* sp. (FJ569890)	100	9E-130	27.6	31.7	27.7	28.2	26.0	27.4	26.2	27.9	25.9	28.8	26.4
						5.7	*2.5*	*1.9*	*2.2*	*1.8*	*2.6*	*3.0*	*2.1*	*1.6*	*1.1*
14	*Actinobacteria*	*Pseudonocardiaceae* sp. (FJ570494)	100	2E-130	21.6	23.3	20.0	23.3	22.3	20.0	14.7	24.2	25.2	22.7	20.5
						5.5	*5.1*	*4.6*	*4.6*	*3.9*	*3.0*	*4.2*	*6.1*	*2.1*	*2.3*
18	*Actinobacteria*	*Acidothermaceae* sp. (JX504954)	100	9E-130	17.9	18.2	16.8	21.2	17.0	18.9	18.0	19.2	14.0	19.4	16.5
						5.5	*6.7*	*7.4*	*6.3*	*7.5*	*7.0*	*9.0*	*5.7*	*3.4*	*3.0*
19	*Gammaproteobacteria*	*Xanthomonadales* sp. (JQ371030)	100	9E-130	17.4	19.8	17.1	16.0	13.8	15.7	18.3	19.3	19.0	17.7	17.1
						2.5	*3.3*	*2.0*	*2.9*	*1.4*	*4.7*	*3.4*	*3.9*	*1.2*	*1.8*
20	*Acidobacteria*	*Acidobacteriaceae* sp. (KC663972)	100	9E-130	13.5	16.0	14.1	14.6	14.6	11.8	12.6	11.6	12.9	13.5	13.5
						2.5	*1.2*	*1.7*	*1.4*	*2.0*	*1.8*	*1.1*	*1.6*	*0.9*	*0.7*
38	*Actinobacteria*	*Acidothermaceae* sp. (FJ661495)	100	9E-130	11.3	12.6	8.8	13.1	10.9	13.2	12.0	11.3	8.6	**12.5**	**10.1**
						3.6	*3.4*	*3.3*	*3.8*	*3.7*	*4.9*	*5.2*	*3.5*	*1.8*	*1.8*
34	*Thermoleophilia*	*Solirubrobacterales* sp. (KC786678)	100	9E-130	10.6	12.4	10.2	12.4	12.4	9.4	9.9	9.1	8.8	10.8	10.3
						2.4	*1.4*	*1.2*	*2.2*	*0.4*	*1.5*	*1.7*	*2.3*	*0.8*	*0.9*
36	*Spartobacteria*	*Chthoniobacterales* sp. (JQ368423)	100	9E-130	9.5	9.9	10.2	9.1	11.6	9.9	10.2	7.7	7.2	9.2	9.8
						1.4	*2.4*	*0.9*	*1.9*	*1.7*	*1.7*	*1.0*	*2.0*	*0.7*	*1.0*
45	*Actinobacteria*	*Acidothermaceae* sp. (GU202348)	100	9E-130	9.4	11.0	8.2	11.2	8.7	9.4	7.8	10.9	7.9	**10.6**	**8.2**
						2.3	*2.2*	*1.7*	*2.3*	*2.0*	*2.4*	*2.8*	*2.0*	*1.0*	*1.0*
29	*Betaproteobacteria*	*Betaproteobacteria* sp. (JQ371661)	100	9E-130	8.4	8.9	9.1	8.8	8.8	8.8	8.4	8.0	6.6	8.6	8.2
						1.1	*1.2*	*1.5*	*1.8*	*1.7*	*1.7*	*1.6*	*1.1*	*0.7*	*0.7*
50	*Alphaproteobacteria*	*Acetobacteraceae* sp. (FJ475432)	100	9E-130	7.6	8.2	8.0	6.6	7.3	4.6	8.2	9.0	8.5	7.1	8.0
						1.8	*1.9*	*0.8*	*1.4*	*1.0*	*2.3*	*2.2*	*2.5*	*0.8*	*1.0*
47	*Sphingobacteriia*	*Chitinophagaceae* sp. (JQ371646)	100	9E-130	7.6	7.5	8.7	5.3	6.5	7.2	8.3	7.2	9.7	**6.8**	**8.3**
						1.7	*1.0*	*1.0*	*0.7*	*1.5*	*1.7*	*1.4*	*1.8*	*0.7*	*0.7*
61	*Spartobacteria*	*Xiphinematobacteraceae* sp. (JQ369593)	99.6	4E-128	7.3	8.4	7.3	7.2	6.0	6.1	6.9	8.6	7.6	7.6	6.9
						1.5	*0.9*	*0.7*	*0.6*	*1.2*	*1.3*	*1.5*	*0.7*	*0.6*	*0.4*
16	*Alphaproteobacteria*	*Xanthobacteraceae* sp. (JN854748)	100	9E-130	7.2	7.6	8.6	6.3	7.8	6.8	7.0	7.2	6.4	7.0	7.5
						1.3	*0.8*	*0.9*	*0.9*	*1.3*	*0.8*	*1.2*	*0.4*	*0.6*	*0.4*

17	*Betaproteobacteria*	*Comamonadaceae* sp. (JQ356919)	100	9E-130	6.8	5.6	6.6	5.6	5.3	5.9	9.1	8.1	8.0	6.3	7.3
						1.0	*1.9*	*1.1*	*0.7*	*1.1*	*1.5*	*1.4*	*1.4*	*0.6*	*0.7*
49	unidentified	*Chloroflexi* sp. (HQ121320)	100	2E-130	6.8	7.0	6.6	10.0	8.5	7.4	6.6	4.2	3.6	7.1	6.4
						1.2	*0.5*	*1.8*	*2.3*	*1.3*	*1.8*	*1.1*	*1.3*	*0.8*	*0.9*
65	*Sphingobacteriia*	*Mucilaginibacter* sp. (HM809499)	100	9E-130	6.7	6.5	5.3	6.9	4.0	7.4	7.0	8.5	8.0	7.3	6.1
						1.0	*0.9*	*1.4*	*1.4*	*1.9*	*1.8*	*1.9*	*1.8*	*0.8*	*0.8*
32	*Actinobacteria*	*Kineosporiaceae* sp. (KC576812)	100	9E-130	6.7	6.2	7.4	6.2	7.3	6.2	4.3	7.5	8.2	6.5	6.8
						1.7	*2.3*	*1.6*	*1.7*	*1.2*	*0.8*	*1.6*	*2.2*	*0.7*	*0.9*
25	*Acidobacteria*	*Acidobacteria* sp. (JQ367877)	100	9E-130	6.2	5.5	6.6	6.2	6.8	6.8	6.4	5.2	6.3	5.9	6.5
						1.0	*0.8*	*0.9*	*1.4*	*0.8*	*1.4*	*0.9*	*1.0*	*0.4*	*0.5*
68	*Actinobacteria*	*Acidothermus* sp. (FJ661423)	99.6	4E-128	6.1	4.8	4.5	7.0	4.3	4.8	9.4	4.5	9.7	5.3	7.0
						2.8	*3.1*	*5.3*	*3.2*	*2.9*	*7.3*	*3.6*	*8.0*	*1.7*	*2.7*
60	*Alphaproteobacteria*	*Methylobacteriaceae* sp. (FJ569009)	99.6	1E-128	6.1	7.0	7.2	7.9	7.0	4.8	4.7	5.8	4.5	6.4	5.8
						0.7	*0.7*	*1.2*	*1.1*	*1.1*	*1.4*	*0.7*	*0.8*	*0.5*	*0.6*
56	*Spartobacteria*	*Chthoniobacterales* sp. (JQ368148)	99.2	2E-126	6.0	5.8	6.4	7.3	7.7	5.7	6.4	4.5	4.4	5.8	6.2
						0.8	*1.1*	*1.0*	*1.4*	*0.9*	*1.5*	*0.8*	*1.0*	*0.5*	*0.7*
74	*Acidobacteria*	*Acidobacterium* sp. (FJ569718)	100	9E-130	5.9	5.9	6.3	4.2	5.1	5.1	7.2	6.0	7.5	5.3	6.5
						1.6	*2.5*	*0.7*	*2.6*	*0.8*	*2.6*	*2.2*	*3.2*	*0.7*	*1.3*
73	*Gammaproteobacteria*	*Xanthomonadales* sp. (JQ371393)	100	9E-130	5.6	4.9	5.4	6.5	5.3	5.6	6.5	5.2	5.1	5.5	5.6
						1.0	*0.7*	*1.2*	*0.8*	*0.8*	*2.0*	*0.7*	*0.6*	*0.5*	*0.6*
31	*Gemmatimonadetes*	*Gemmatimonadaceae* sp. (JN854741)	100	9E-130	5.6	5.7	6.8	6.5	6.1	6.0	5.5	4.4	3.4	5.7	5.5
						1.1	*1.2*	*0.8*	*1.1*	*1.0*	*1.1*	*1.1*	*0.8*	*0.5*	*0.6*
37	*Betaproteobacteria*	*Nitrosomonadaceae* sp. (HQ120221)	100	9E-130	5.0	4.8	5.9	5.5	5.5	4.7	5.2	4.1	4.2	4.8	5.2
						1.0	*1.1*	*0.7*	*0.7*	*0.7*	*1.1*	*0.6*	*0.6*	*0.4*	*0.4*
62	*Acidobacteria*	*Acidobacteriaceae* sp. (JN850508)	100	9E-130	4.9	5.2	4.8	4.6	4.9	6.0	5.2	4.3	4.6	5.0	4.9
						1.0	*0.5*	*1.4*	*0.4*	*1.9*	*1.6*	*0.5*	*0.4*	*0.6*	*0.4*

WET TUNDRA

						Relative abundance (‰)									
OTU	Class	Best identified hit (accession number)	Similarity (%)	E-value	Abundance (‰)	June C	June S	July C	July S	Sept C	Sept S	Octo C	Octo S	SA C	SA S
1	*Spartobacteria*	*Chthoniobacterales* sp. (JQ368031)	100	9E-130	48.4	43.9	45.8	51.8	53.5	50.9	50.9	43.0	47.6	47.4	49.4
						4.2	*7.1*	*6.7*	*6.9*	*4.7*	*6.7*	*2.6*	*2.5*	*2.4*	*2.9*
10	unidentified	*Chloroflexi* sp. (HM270099)	100	9E-130	22.3	24.6	24.7	25.4	25.7	19.0	20.2	19.2	19.6	22.1	22.6
						2.0	*2.5*	*2.0*	*1.6*	*1.3*	*1.9*	*0.9*	*1.0*	*1.0*	*1.0*
15	*Actinobacteria*	*Intrasporangiaceae* sp. (JX981785)	100	9E-130	16.4	21.5	17.7	22.2	16.7	15.0	10.9	13.6	13.4	18.1	14.7
						4.5	*4.7*	*5.1*	*4.9*	*2.2*	*3.1*	*1.0*	*1.9*	*1.9*	*1.9*
21	*Betaproteobacteria*	*Nitrosomonadaceae* sp. (JQ371214)	100	9E-130	12.8	14.4	11.1	12.0	10.1	14.7	13.1	14.5	12.3	13.9	11.7
						0.8	*1.1*	*1.4*	*1.3*	*1.1*	*1.7*	*1.2*	*0.4*	*0.6*	*0.6*
30	*Holophagae*	*Holophagae* sp. (JQ367705)	100	9E-130	12.2	16.2	14.7	18.6	12.2	12.5	6.0	9.3	8.4	14.1	10.3
						3.0	*3.9*	*1.0*	*2.1*	*2.8*	*2.1*	*1.3*	*0.9*	*1.3*	*1.4*
24	*Actinobacteria*	*Nakamurellaceae* sp. (JX667928)	100	9E-130	11.3	12.4	12.4	13.2	11.5	9.8	9.9	10.7	10.5	11.5	11.1
						1.6	*2.0*	*2.1*	*1.6*	*0.8*	*1.2*	*1.0*	*0.9*	*0.7*	*0.7*
23	*Acidobacteria*	*Acidobacteria* sp. (HQ153207)	100	2E-130	10.8	7.8	11.9	8.9	11.2	12.0	12.4	10.3	11.9	**9.7**	**11.9**
						0.5	*1.8*	*0.6*	*1.6*	*0.6*	*0.7*	*1.3*	*1.3*	*0.5*	*0.7*
16	*Alphaproteobacteria*	*Xanthobacteraceae* sp. (JN854748)	100	9E-130	10.0	12.1	11.1	11.4	10.6	9.6	8.9	7.3	9.2	10.1	10.0
						0.9	*1.3*	*1.3*	*1.1*	*1.1*	*0.8*	*0.3*	*0.6*	*0.6*	*0.5*

26	*Acidobacteria*	*Acidobacteria* sp. (FJ568507)	99.6	1E-128	9.9	9.0<br>*0.9*	9.7<br>*1.0*	8.8<br>*1.2*	8.6<br>*1.0*	10.2<br>*0.8*	11.8<br>*0.7*	10.7<br>*0.7*	10.1<br>*0.9*	9.7<br>*0.5*	10.0<br>*0.5*
17	*Betaproteobacteria*	*Comamonadaceae* sp. (JQ356919)	100	9E-130	9.6	9.6<br>*1.4*	11.4<br>*1.5*	9.8<br>*0.9*	8.1<br>*0.5*	7.6<br>*1.2*	9.3<br>*1.0*	10.7<br>*1.1*	10.5<br>*1.2*	9.4<br>*0.6*	9.8<br>*0.6*
27	*Holophagae*	*Holophagae* sp. (JX967660)	100	9E-130	9.4	10.0<br>*1.2*	9.5<br>*1.2*	12.8<br>*1.8*	11.3<br>*1.3*	7.7<br>*0.9*	6.9<br>*1.0*	8.8<br>*0.8*	8.4<br>*0.8*	9.8<br>*0.7*	9.0<br>*0.6*
3	*Alphaproteobacteria*	*Bradyrhizobium* sp. (JQ357060)	100	9E-130	9.3	8.9<br>*0.7*	9.7<br>*0.9*	8.4<br>*0.6*	9.4<br>*0.9*	9.0<br>*1.1*	9.5<br>*1.6*	9.6<br>*1.1*	10.1<br>*1.1*	9.0<br>*0.4*	9.7<br>*0.5*
28	*Betaproteobacteria*	*Nitrosomonadaceae* sp. (EF018556)	100	2E-130	9.3	9.4<br>*0.8*	8.4<br>*1.1*	9.0<br>*0.9*	9.7<br>*1.2*	9.9<br>*0.9*	8.8<br>*1.1*	9.9<br>*0.4*	9.3<br>*0.8*	9.6<br>*0.4*	9.1<br>*0.5*
39	*Betaproteobacteria*	*Nitrosomonadaceae* sp. (KC605641)	100	9E-130	8.3	11.4<br>*1.8*	7.3<br>*1.6*	10.3<br>*1.5*	6.8<br>*0.9*	8.8<br>*2.3*	6.4<br>*1.7*	8.4<br>*0.7*	7.5<br>*0.9*	9.7<br>*0.8*	7.0<br>*0.6*
40	unidentified	*Acidobacteria* sp. (FJ655938)	100	9E-130	8.0	9.9<br>*1.4*	7.6<br>*0.7*	9.2<br>*1.1*	8.8<br>*0.8*	6.7<br>*0.6*	6.6<br>*0.8*	7.8<br>*0.5*	7.2<br>*0.2*	8.4<br>*0.5*	7.6<br>*0.4*
25	*Acidobacteria*	*Acidobacteria* sp. (JQ367877)	100	9E-130	7.8	7.2<br>*0.8*	8.2<br>*0.9*	8.6<br>*0.8*	9.7<br>*0.6*	6.9<br>*0.6*	8.7<br>*0.4*	6.1<br>*0.4*	7.4<br>*0.3*	**7.2**<br>*0.4*	**8.5**<br>*0.3*
41	*Nitrospira*	*Nitrospiraceae* sp. (JQ372043)	100	9E-130	7.7	7.3<br>*1.0*	6.9<br>*1.4*	7.4<br>*0.9*	8.9<br>*0.8*	7.8<br>*0.9*	8.2<br>*1.3*	7.4<br>*1.0*	7.9<br>*0.7*	7.5<br>*0.4*	8.0<br>*0.5*
43	*Sphingobacteriia*	*Chitinophagaceae* sp. (KC255350)	100	9E-130	7.2	6.4<br>*0.9*	6.9<br>*0.7*	6.1<br>*0.6*	5.5<br>*0.7*	9.3<br>*1.5*	9.1<br>*1.0*	7.3<br>*0.8*	7.1<br>*0.5*	7.3<br>*0.5*	7.2<br>*0.4*
31	*Gemmatimonadetes*	*Gemmatimonadaceae* sp. (JN854741)	100	9E-130	6.9	7.0<br>*0.6*	5.5<br>*1.7*	10.9<br>*1.9*	8.8<br>*1.8*	5.1<br>*0.7*	4.9<br>*1.6*	7.1<br>*1.4*	6.2<br>*0.8*	7.5<br>*0.7*	6.3<br>*0.8*
44	*Alphaproteobacteria*	*Rhizobiales* sp. (FJ568560)	100	9E-130	6.7	7.7<br>*0.6*	5.8<br>*0.4*	6.9<br>*0.7*	7.1<br>*0.6*	6.4<br>*0.8*	6.5<br>*0.7*	6.5<br>*0.8*	6.4<br>*0.3*	6.9<br>*0.3*	6.4<br>*0.3*
29	*Betaproteobacteria*	*Betaproteobacteria* sp. (JQ371661)	100	9E-130	6.3	6.7<br>*0.9*	7.4<br>*1.8*	8.5<br>*1.6*	7.4<br>*1.6*	5.4<br>*0.8*	4.1<br>*1.3*	5.2<br>*0.6*	5.7<br>*0.7*	6.5<br>*0.6*	6.1<br>*0.7*
55	*Alphaproteobacteria*	*Rhizobiales* sp. (KC683078)	100	9E-130	6.2	5.7<br>*1.1*	5.7<br>*0.9*	6.6<br>*1.2*	6.7<br>*0.9*	6.2<br>*1.3*	6.6<br>*0.6*	5.8<br>*0.5*	5.9<br>*1.1*	6.1<br>*0.5*	6.2<br>*0.4*
51	*Betaproteobacteria*	*Betaproteobacteria* sp. (FR749807)	99.6	2E-126	6.0	5.8<br>*0.5*	5.8<br>*0.5*	7.4<br>*0.8*	7.9<br>*1.0*	5.0<br>*0.3*	5.4<br>*0.8*	5.4<br>*0.5*	5.4<br>*0.5*	5.9<br>*0.3*	6.1<br>*0.4*
32	*Actinobacteria*	*Kineosporiaceae* sp. (KC576812)	100	9E-130	5.9	5.5<br>*0.5*	5.3<br>*0.7*	6.2<br>*0.7*	5.4<br>*0.5*	6.4<br>*0.7*	5.5<br>*0.5*	6.8<br>*0.5*	5.7<br>*0.2*	6.2<br>*0.3*	5.5<br>*0.2*
66	*Nitrospira*	*Nitrospirales* sp. (JX981781)	100	9E-130	5.8	5.8<br>*1.0*	7.0<br>*2.6*	8.4<br>*1.0*	5.5<br>*1.2*	7.2<br>*1.7*	4.2<br>*2.2*	4.5<br>*1.1*	4.2<br>*0.8*	6.5<br>*0.7*	5.2<br>*0.9*
37	*Betaproteobacteria*	*Nitrosomonadaceae* sp. (HQ120221)	100	9E-130	5.8	6.2<br>*0.8*	6.0<br>*0.4*	6.0<br>*0.5*	6.4<br>*0.8*	6.6<br>*0.7*	4.9<br>*0.3*	5.6<br>*0.5*	4.8<br>*0.6*	6.1<br>*0.3*	5.5<br>*0.3*
48	*Thermoleophilia*	*Solirubrobacteraceae* sp. (KC554684)	100	9E-130	5.5	5.6<br>*1.3*	6.2<br>*1.4*	4.4<br>*0.8*	7.4<br>*1.6*	4.2<br>*0.4*	6.4<br>*1.4*	4.1<br>*0.4*	5.7<br>*0.7*	**4.6**<br>*0.4*	**6.4**<br>*0.6*
46	*Alphaproteobacteria*	*Bradyrhizobiaceae* sp. (JF437363)	100	9E-130	5.4	5.0<br>*0.5*	5.5<br>*0.9*	4.1<br>*0.4*	4.7<br>*0.7*	6.0<br>*0.7*	7.2<br>*1.2*	5.6<br>*0.6*	5.3<br>*0.4*	5.2<br>*0.3*	5.7<br>*0.4*
52	*Betaproteobacteria*	*Betaproteobacteria* sp. (JQ372120)	100	9E-130	5.4	6.2<br>*0.8*	5.0<br>*0.9*	5.8<br>*0.6*	4.4<br>*0.5*	5.8<br>*0.7*	5.0<br>*0.6*	5.7<br>*0.5*	5.4<br>*0.3*	5.9<br>*0.3*	5.0<br>*0.3*
72	*Betaproteobacteria*	*Nitrosomonadaceae* sp. (JX986326)	100	9E-130	5.2	5.1<br>*0.5*	5.2<br>*0.8*	4.4<br>*0.4*	4.3<br>*0.2*	5.3<br>*1.0*	6.1<br>*0.7*	5.5<br>*0.8*	5.4<br>*0.6*	5.1<br>*0.3*	5.2<br>*0.3*
