# Supplementary material for: Fast response of fungal and prokaryotic communities to climate change manipulation in two contrasting tundra soils
Source: Environ Microbiome. 2019 Sep 18;14:6. doi: 10.1186/s40793-019-0344-4 (PMC7989089; doi:10.1186/s40793-019-0344-4)

Additional file 9

Relative abundance of archaea in prokaryotic sequence pool in dry (A) and wet (B) tundra soil in control (green) and snow-manipulated (red) plots by season and as a seasonal average (SA). The data represent the means with standard errors (n=6), for seasonal average (n=24).

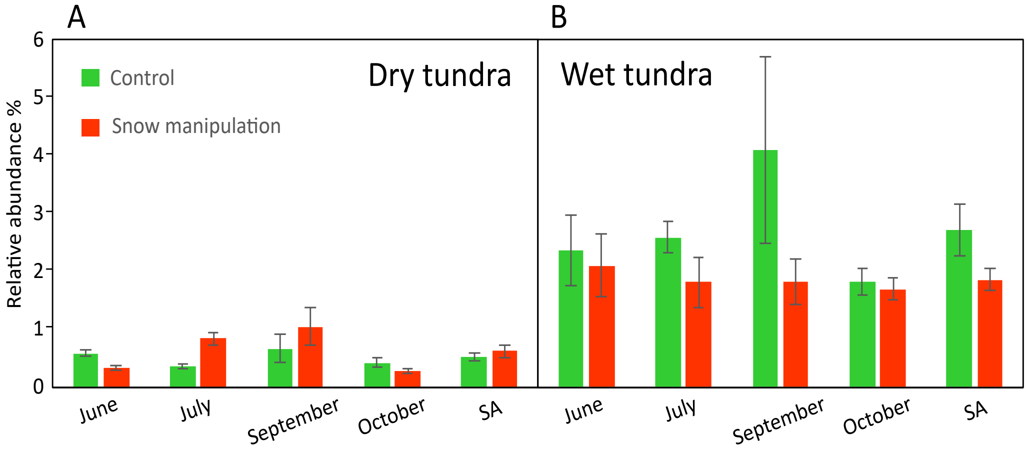

Supplement: Supplementary file 9 — Relative abundance of archaea in prokaryotic sequence pool in dry (A) and wet (B) tundra soil in control (green) and snow-manipulated (red) plots by season and as a seasonal average (SA). The data represent the means with standard errors (n = 6), for seasonal average (n = 24). (PDF 57 kb) [file 40793_2019_344_MOESM9_ESM.pdf]
